# Reduced FBXO22 skews human trophoblast fate equilibrium toward syncytialization via polyubiquitinating the CoREST complex

**DOI:** 10.1093/nar/gkag557

**Published:** 2026-06-04

**Authors:** Hongli Li, Guangmin Song, Linwei Zhou, Man Zhang, Yun Li, Xinmi Liu, Xing Wang, Li Yang, Xinyi Tao, Richard D Cannon, Richard Saffery, Boris Novakovic, Hongbo Qi, Xiaobo Zhou, Hua Zhang

**Affiliations:** Department of Obstetrics and Gynecology, Chongqing Key Laboratory of Maternal and Fetal Medicine/Joint International Research Laboratory of Reproduction & Development, Ministry of Education/The Innovation and Talent Recruitment Base of Maternal-Fetal Medicine, The First Affiliated Hospital of Chongqing Medical University, No. 1 Youyi Rd, Yuzhong District, Chongqing 400016, China; Department of Obstetrics and Gynecology, Chongqing Key Laboratory of Maternal and Fetal Medicine/Joint International Research Laboratory of Reproduction & Development, Ministry of Education/The Innovation and Talent Recruitment Base of Maternal-Fetal Medicine, The First Affiliated Hospital of Chongqing Medical University, No. 1 Youyi Rd, Yuzhong District, Chongqing 400016, China; Department of Obstetrics and Gynecology, Chongqing Key Laboratory of Maternal and Fetal Medicine/Joint International Research Laboratory of Reproduction & Development, Ministry of Education/The Innovation and Talent Recruitment Base of Maternal-Fetal Medicine, The First Affiliated Hospital of Chongqing Medical University, No. 1 Youyi Rd, Yuzhong District, Chongqing 400016, China; Department of Obstetrics and Gynecology, Chongqing Key Laboratory of Maternal and Fetal Medicine/Joint International Research Laboratory of Reproduction & Development, Ministry of Education/The Innovation and Talent Recruitment Base of Maternal-Fetal Medicine, The First Affiliated Hospital of Chongqing Medical University, No. 1 Youyi Rd, Yuzhong District, Chongqing 400016, China; Department of Obstetrics and Gynecology, Chongqing Key Laboratory of Maternal and Fetal Medicine/Joint International Research Laboratory of Reproduction & Development, Ministry of Education/The Innovation and Talent Recruitment Base of Maternal-Fetal Medicine, The First Affiliated Hospital of Chongqing Medical University, No. 1 Youyi Rd, Yuzhong District, Chongqing 400016, China; Department of Obstetrics and Gynecology, Chongqing Key Laboratory of Maternal and Fetal Medicine/Joint International Research Laboratory of Reproduction & Development, Ministry of Education/The Innovation and Talent Recruitment Base of Maternal-Fetal Medicine, The First Affiliated Hospital of Chongqing Medical University, No. 1 Youyi Rd, Yuzhong District, Chongqing 400016, China; Department of Obstetrics and Gynecology, Chongqing Key Laboratory of Maternal and Fetal Medicine/Joint International Research Laboratory of Reproduction & Development, Ministry of Education/The Innovation and Talent Recruitment Base of Maternal-Fetal Medicine, The First Affiliated Hospital of Chongqing Medical University, No. 1 Youyi Rd, Yuzhong District, Chongqing 400016, China; Department of Obstetrics and Gynecology, Chongqing Key Laboratory of Maternal and Fetal Medicine/Joint International Research Laboratory of Reproduction & Development, Ministry of Education/The Innovation and Talent Recruitment Base of Maternal-Fetal Medicine, The First Affiliated Hospital of Chongqing Medical University, No. 1 Youyi Rd, Yuzhong District, Chongqing 400016, China; Department of Obstetrics and Gynecology, Chongqing Key Laboratory of Maternal and Fetal Medicine/Joint International Research Laboratory of Reproduction & Development, Ministry of Education/The Innovation and Talent Recruitment Base of Maternal-Fetal Medicine, The First Affiliated Hospital of Chongqing Medical University, No. 1 Youyi Rd, Yuzhong District, Chongqing 400016, China; Department of Oral Sciences, Sir John Walsh Research Institute, Faculty of Dentistry, University of Otago, Dunedin 9016, New Zealand; Molecular Immunity, Infection and Immunity Theme, Murdoch Children’s Research Institute, Royal Children’s Hospital, Parkville, VIC 3052, Australia; Molecular Immunity, Infection and Immunity Theme, Murdoch Children’s Research Institute, Royal Children’s Hospital, Parkville, VIC 3052, Australia; Department of Obstetrics and Gynecology, Chongqing Key Laboratory of Maternal and Fetal Medicine/Joint International Research Laboratory of Reproduction & Development, Ministry of Education/The Innovation and Talent Recruitment Base of Maternal-Fetal Medicine, The First Affiliated Hospital of Chongqing Medical University, No. 1 Youyi Rd, Yuzhong District, Chongqing 400016, China; Department of Obstetrics and Gynecology, Chongqing Key Laboratory of Maternal and Fetal Medicine/Joint International Research Laboratory of Reproduction & Development, Ministry of Education/The Innovation and Talent Recruitment Base of Maternal-Fetal Medicine, The First Affiliated Hospital of Chongqing Medical University, No. 1 Youyi Rd, Yuzhong District, Chongqing 400016, China; Department of Obstetrics and Gynecology, Chongqing Key Laboratory of Maternal and Fetal Medicine/Joint International Research Laboratory of Reproduction & Development, Ministry of Education/The Innovation and Talent Recruitment Base of Maternal-Fetal Medicine, The First Affiliated Hospital of Chongqing Medical University, No. 1 Youyi Rd, Yuzhong District, Chongqing 400016, China

## Abstract

The precise balance between human trophoblast stem cells (hTSCs) self-renewal and differentiation into syncytiotrophoblasts (STBs) is essential for proper placental development. While the transcriptional and signaling networks regulating this process have been extensively studied, the contribution of protein homeostasis remains poorly understood. Here, we identify FBXO22, the substrate recognition subunit of the SCF E3 ubiquitin ligase complex, as a key regulator of trophoblast fate. We found that FBXO22 was enriched in the nuclei of cytotrophoblasts (CTBs) and levels were reduced markedly in early placental villi from patients with recurrent pregnancy loss (RPL). Experimental loss of FBXO22 compromised hTSC stemness and led to aberrant premature differentiation toward STBs. Mechanistically, FBXO22 selectively ubiquitinates and destabilizes the CoREST complex, thereby coordinating with HDAC1 and LSD1 to regulate histone modifications, including H3K27 acetylation (H3K27ac) and H3K9 dimethylation (H3K9me2). Disruption of this nuclear ubiquitination pathway perturbs the balance between proliferation and differentiation, ultimately impairing placental development. Our findings uncover a previously unrecognized nuclear role of FBXO22 in maintaining cellular homeostasis, linking ubiquitin-mediated protein degradation to trophoblast fate determination and providing new insights into the molecular pathology underlying early pregnancy loss.

## Graphical Abstract

**Graphical Abstract:**
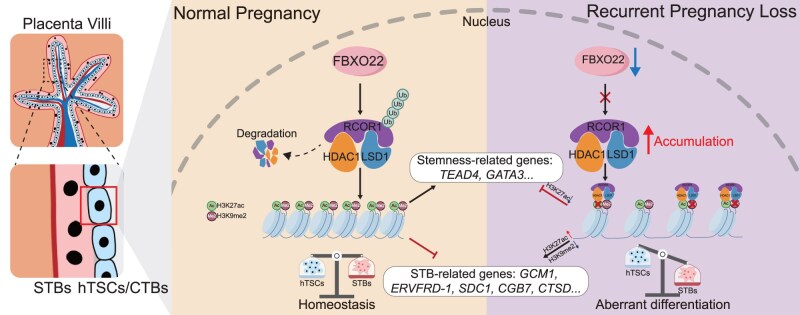

## Introduction

Recurrent pregnancy loss (RPL), defined as two or more consecutive pregnancy failures before 20 weeks of gestation [1, 2], remains a significant clinical challenge in reproductive medicine. Its etiological factors include anatomic abnormalities, genetic defects, and immune or endocrine dysfunctions [3]. Increasing evidence implicates the placenta, a unique and transient organ mediating nutrient exchange and endocrine signaling between the mother and the fetus, as a critical determinant of pregnancy outcome [4, 5]. Abnormal placental development or dysfunction can lead to miscarriage, intrauterine growth restriction, and even stillbirth [6]. Despite the critical role for trophoblast self-renewal and differentiation in early placental development, the molecular mechanisms controlling these processes are not fully understood.

Placental development begins at the blastocyst stage of embryo formation with the trophectoderm (TE), which subsequently differentiates into a highly specialized multinucleated structure, the placenta [4, 7]. The function of the human placenta is primarily carried out by three primary trophoblast cell types: syncytiotrophoblasts (STBs), extravillous trophoblasts (EVTs), and cytotrophoblasts (CTBs) [8, 9]. CTBs, appearing eight days after fertilization, act as multipotent progenitors capable of differentiating into both STBs and EVTs. Human trophoblast stem cells (hTSCs) can provide a powerful *in vitro* model to study placental development, as they can differentiate into both STBs and EVTs and form placental-like organoid structures [10, 11]. Epigenetic mechanisms, particularly histone modifications, play pivotal roles in directing the fate of hTSCs [12–14]. Such modifications can establish either transcriptionally repressive or permissive chromatin states, thereby influencing trophoblast lineage specification [15, 16]. For example, lysine-specific demethylase 1 (LSD1) promotes syncytialization by removing H3K9me2 methylation at promoters of STB-related genes and cooperating with the transcription factor GATA2 to recruit RNA polymerase II [17]. Similarly, global histone H3 acetylation undergoes dynamic changes during the transition from CTBs to STBs [18, 19]. Nevertheless, the upstream regulatory mechanisms controlling these nuclear-localized chromatin-modifying enzymes in trophoblast biology are still poorly defined.

In addition to epigenetic regulation, post-translational modifications such as ubiquitination add a further layer of control. Ubiquitination directs particular proteins for proteasomal degradation and regulates diverse cellular processes including growth, proliferation, and differentiation [20]. This process is mediated by E3 ubiquitin ligase complexes, which confer substrate specificity via their accessory subunits. The SKP1 CUL1-F-box (SCF)-type E3 ubiquitin ligase is a prototypical RING-type E3 ubiquitin ligase, for which F-box proteins act as interchangeable substrate-recognition modules [21]. FBXO22, one such FBXO protein [22], has been shown to regulate diverse biological processes by ubiquitination of substrates such as BACH1, mTORC1, KDM5A, TP53, and PTEN [23–27]. Importantly, Rull *et al*. reported that FBXO22 expression is markedly reduced in villous tissues from RPL patients [28]. Yet, the functional significance of FBXO22 in trophoblast biology and its relevance to RPL remain unknown.

In this study we identify FBXO22 as a nuclear E3 ubiquitin ligase essential for trophoblast fate balance. Employing *in vivo* and *in vitro* models, we demonstrate that FBXO22 is enriched in the nuclei of CTBs and regulates hTSC self-renewal and syncytialization through stabilization of the CoREST complex, and modulation of H3K9me2 and H3K27ac modifications. Loss of *FBXO22* disrupts these epigenetic programs, driving aberrant syncytialization and impaired placental development. Notably, we uncover an association between reduced FBXO22 expression, dysregulated CoREST complex activity, and RPL. Together, these findings establish FBXO22 as a nuclear E3 ubiquitin ligase critical for maintaining trophoblast lineage homeostasis and suggest that its dysregulation may contribute to the molecular pathology of RPL.

## Materials and methods

### Human samples

This study was performed following the principles of the Declaration of Helsinki and approved by the Medical Ethics Committee of the First Affiliated Hospital of Chongqing Medical University, Chongqing, China (No. 2021–746). All participants provided informed consent and RPL participants underwent elective termination of pregnancy for medical reasons in compliance with legal and ethical standards. After collecting early placental villi tissue, the dissected samples were rinsed with physiological saline and immediately frozen at −80°C for further analysis. The definition of RPL followed the criteria established by the European Society of Human Reproduction and Embryology (ESHRE)—two or more consecutive clinically confirmed pregnancy failures [29, 30]. Patients with uterine anatomical abnormalities, immune disorders, fetal chromosomal abnormalities, endocrine or metabolic diseases, or infectious diseases were excluded. The control group consisted of women without a history of miscarriage who underwent elective termination of normal pregnancies for non-medical reasons. Inclusion criteria for the normal middle and late pregnancy group were as follows: absence of pregnancy complications or chronic diseases, placental weight appropriate for gestational age, and intact placental morphology. Detailed clinical information for participants who provided early placental villous samples is presented in Supplementary Table S1.

### hTSC derivation, culture, and differentiation

hTSCs were cultured and differentiated into STBs and EVTs following previously published protocols [10, 13, 31]. Briefly, hTSCs were isolated from fresh human chorionic villus tissue and cultured in six-well microtiter plates pre-coated with collagen IV (5 μg/ml, 354233, Corning). Cells were cultured in hTSC medium consisting of DMEM/F12 (L310KJ, BasalMedia) supplemented with 1% penicillin–streptomycin (PS, 15140122, ThermoFisher), 0.2% FBS (10099 141, ThermoFisher), 0.1 mM 2-mercaptoethanol (21985023, ThermoFisher), 0.3% BSA (A9418, Sigma), 1% ITS-X supplement (S452J7, BasalMedia), 1.5 μg/ml L-ascorbic acid (A5960, Sigma), 50 ng/ml EGF (315-09-100, PeproTech), 5 μM Y-27632 (S1049, Selleck), 2 μM CHIR99021 (S1263, Selleck), 0.5 μM A83-01 (HY-10432A, MedChemExpress), 1 μM SB431542 (HY-10431, MedChemExpress), and 0.8 mM VPA (HY-10585, MedChemExpress), and incubated at 37°C in a humidified atmosphere of 5% CO_2_. Cells were passaged at 60%–80% confluency using TrypLE Express (12604021, ThermoFisher). For STB differentiation in 2D, hTSCs were seeded on dishes coated with 2.5 μg/ml collagen IV and cultured in STB (2D) medium, consisting of DMEM/F12 supplemented with 0.5% PS, 0.1 mM 2-mercaptoethanol, 0.3% BSA, 1% ITS-X supplement, 2.5 μM Y-27632, 2 μM Forskolin (FSK, HY-15371, MedChemExpress), and 4% KSR (10828010, ThermoFisher). hTSCs were routinely subjected to STB induction for 72 h. For STB differentiation in 3D, 50 ng/ml EGF was included in the medium. For EVT differentiation, hTSCs were seeded on dishes pre-coated with 1 μg/ml collagen IV and cultured in EVT differentiation medium, composed of DMEM/F12 supplemented with 0.1 mM 2-mercaptoethanol, 0.5% PS, 0.3% BSA, 1% ITS-X supplement, 100 ng/ml NRG1 (26941, CST), 5 μM A83-01, 2.5 μM Y-27632, 4% KSR, and 2% Matrigel (356231, Corning). After 3 days, the medium was replaced with EVT medium lacking NRG1 and containing 0.5% Matrigel for a further 3 days. On day 6, cells were dissociated with TrypLE Express and subsequently cultured for an additional 2–3 days in EVT medium without NRG1 or KSR, but supplemented with 0.5% Matrigel.

### Human trophoblast organoid derivation and culture

For trophoblast organoid (TO) derivation [32, 33], approximately 5 × 10^3^ hTSCs were embedded in Matrigel droplets and cultured at 37°C in trophoblast organoid medium (TOM), composed of Advanced DMEM/F12 (12634010, ThermoFisher) supplemented with N2 supplement (17502048, ThermoFisher), B27 supplement (17504044, ThermoFisher), 1% PS, 1.25 mM *N*-acetyl-L-cysteine (A9165, Merck), 2 mM L-glutamine (25030081, ThermoFisher), 50 ng/ml recombinant human EGF, 1.5 μM CHIR99021, 80 ng/ml recombinant human R-spondin-1 (HY-P7114, MedChemExpress), 100 ng/ml recombinant human FGF-2 (10018, PeproTech), 50 ng/ml recombinant human HGF (10039, PeproTech), 500 nM A83-01, 2.5 μM prostaglandin E2 (HY101952, MedChemExpress), and 2.5 μM Y-27632. TOM was replaced every 2 days, and organoids were collected on day 7 for subsequent analyses.

### Cell cultures

BeWo cells (CL-0500, Procell) were maintained in DMEM/F12 medium supplemented with 10% FBS (A5256701, ThermoFisher) and 1% PS. For the cell fusion assay, cells were treated with 50 μM FSK for 48–72 h. HEK293T cells (CRL-11268, ATCC) were cultured in high-glucose DMEM (11995073, ThermoFisher) containing 10% FBS and 1% PS (15140122, ThermoFisher). All cell lines were free from mycoplasma contamination and kept in a humidified atmosphere containing 5% CO_2_ at 37°C.

### Reagents and antibodies

The following reagents were used in this study: HDAC inhibitor Romidepsin (S3020, Selleck), and LSD1 inhibitor GSK-LSD1 (S7574, Selleck). Comprehensive information on the chemicals and antibodies used in this study is provided in Supplementary Table S2.

### Plasmid construction, siRNA transfection, and lentiviral infection

Overexpression plasmids for FBXO22 (pCDNA3.1-FBXO22-3 × HA), HDAC1 (pEnCMV-HDAC1-3 × FLAG), RCOR1 (pCMV-RCOR1-3 × Myc), LSD1 (pCDNA3.1-LSD1-V5 and pCDNA3.1-LSD1-V5-TurboID), ubiquitin (pCDNA3.1–3 × Myc-Ub, pCDNA3.1–3 × Myc-Ub-K48R, pCDNA3.1–3 × Myc-Ub-K63R, pCMV-His-Ub, pCMV-His-Ub-K48R, and pCMV-His-Ub-K63R), CUL1 (pCMV-CUL1-3 × FLAG), SKP1 (pCMV-SKP1-3 × FLAG), and RBX1 (pCMV-RBX1-3 × FLAG) were either constructed by MiaoLingbio (Wuhan, China) or generated in our laboratory. Plasmid transfection of HEK293T cells was performed using Lipofectamine 3000 (Invitrogen). Negative control siRNA (si-*NC*) and FBXO22-targeting siRNA (si-*FBXO22*) were purchased from RiboBio (Guangzhou, China). The si-*FBXO22* sequence was 5′-GCACCTTCGTGTTGAGTAA-3′. siRNA transfection of hTSCs was carried out using Lipofectamine RNAiMAX (Invitrogen) at a final concentration of 20 nM. The *FBXO22* overexpression lentivirus (HBLV-h-*FBXO22*-3 × HA-ZsGreen) and *FBXO22* knockdown lentivirus (HBLV-h-*FBXO22* shRNA) were purchased from HanBio (Shanghai, China). Lentiviral transduction of hTSCs was performed in the presence of polybrene.

### Immunoprecipitation (IP) and mass spectrometry

Protein extraction from cultured hTSCs, protein concentration determination, and immunoprecipitation were performed as described previously [34]. Briefly, cells were lysed in IP lysis buffer (87787, ThermoFisher) containing protease and phosphatase inhibitors (B14001 and B15001, Selleck). Cell lysates were incubated with primary antibodies at 4°C overnight. Protein A/G beads (20421, ThermoFisher) were then added and incubated for 6 h, followed by five washes with IP buffer. Proteins were eluted by boiling beads in SDS–PAGE sample buffer, and the eluates were subjected to liquid chromatography-mass spectrometry (Novogene, China), silver staining (P0017S, Beyotime), and immunoblotting analyses.

### Immunoblot analysis

For immunoblot analysis, cell culture and tissue protein samples were lysed in RIPA buffer supplemented with protease and phosphatase inhibitors, followed by a 30 min incubation on ice. Protein concentration was determined using a BCA protein assay kit according to the manufacturer’s instructions (P0009, Beyotime). For nuclear and cytoplasmic fractionation, cells were processed following the manufacturer’s instructions (OP-0002, EpigenTek). Briefly, cells were resuspended in NE1 buffer and incubated on ice for 10 min, followed by vortexing and centrifugation to obtain the cytoplasmic extract (supernatant) and nuclear fraction (pellet). The nuclear pellet was then lysed in NE2 buffer, incubated on ice for 15 min, and centrifuged to collect the nuclear protein extract. Protein samples were separated by 8%–13% SDS–PAGE and transferred onto PVDF membranes using a wet transfer system. Membranes were blocked with 5% non-fat milk in TBST for 1 h at room temperature, then incubated with specific primary antibodies at 4°C overnight. Antibodies used included: anti-FBXO22 (1:1000, Proteintech), anti-LaminB1 (1:1000, Proteintech), anti-β-Catenin (1:1000, Proteintech), anti-HDAC1 (1:1000, CST), anti-RCOR1 (1:1000, ABclonal), anti-LSD1 (1:2000, ABclonal), anti-TEAD4 (1:1000, GeneTex), anti-GCM1 (1:500, Proteintech), anti-CGB (1:2000, ThermoFisher), anti-H3 (1:1000, CST), anti-H3K27ac (1:1000, CST), anti-H3K9me2 (1:1000, Active Motif), anti-H3K4me2 (1:2000, Abcam), anti-GAPDH (1:1000, Proteintech), anti-β-actin (1:2000, Proteintech), anti-HA (1:2000, Proteintech), anti-Flag (1:2000, Proteintech), anti-Myc (1:1000, ABclonal), anti-V5 (1:1000, Proteintech), anti-His (1:500, CST), and anti-Cytokeratin 7 (1:1000, Proteintech). After washing with TBST, membranes were incubated for 1 h at room temperature with HRP-conjugated secondary antibodies (1:5000, ABclonal), mouse anti-rabbit IgG (Conformation Specific) (1:1000, CST), or universal secondary antibody for avoiding heavy and light chains (1:1000, Abmart). Protein bands were visualized using enhanced chemiluminescence (ECL) reagents (K-12045-D50, Advansta), detected with chemiluminescence imaging systems (Bio-Rad FusionFx), and quantified using ImageJ software. Relative expression of target proteins was normalized to housekeeping proteins (H3, GAPDH, or β-actin) on the same membrane. For proteins with similar molecular weights, membranes were incubated with Restore Western Blot Stripping Buffer (21059, ThermoFisher) as recommended by the manufacturer to remove previously bound antibodies, and then re-probed with different primary antibodies for subsequent immunoblotting.

### Protein stability and degradation assay

For the assay of CoREST complex degradation, *FBXO22*-overexpressing hTSCs were treated with the proteasome inhibitor MG132 (20 μM, S1748, MedChemExpress) or the autophagy inhibitor 3-MA (20 mM, HY-19312, MedChemExpress) for 6 h, with DMSO-treated cells serving as the negative control. To assess the protein stability of the CoREST complex, *FBXO22*-knockdown and control hTSCs were treated with the protein synthesis inhibitor cycloheximide (CHX, 20 μg/ml) for 0, 4, 8, or 12 h, followed by protein extraction and immunoblotting analysis.

### CoREST complex ubiquitylation assay

MG132 (20 μM, S1748, MedChemExpress) was added to the culture medium 6 h prior to cell collection. Target proteins in the cell lysates were immunoprecipitated using specific primary antibodies. Proteins were eluted by boiling the beads in SDS–PAGE sample buffer, and ubiquitination was analyzed by immunoblotting with specific antibodies.

### Reconstitution and purification of the SCF^FBXO22^ ubiquitin ligase complex

Based on a previously reported protocol [35], 293T cells were transfected with plasmids encoding the components of the SCF^FBXO22^ complex (including HA-FBXO22, Flag-CUL1, Flag-SKP1, and Flag-RBX1), then harvested and lysed in lysis buffer containing 50 mM Tris–HCl (pH 7.5), 150 mM NaCl, 0.5% NP-40, 10% glycerol, and 1× protease inhibitor cocktail. After clarification by centrifugation, the soluble fractions were incubated with anti-HA magnetic beads (88836, ThermoFisher) at 4°C overnight. The beads were subsequently washed three times sequentially with buffer A (15 mM Tris–HCl, pH 7.4, 500 mM NaCl, and 0.25% NP-40) and buffer B (25 mM Tris–HCl, pH 7.5, 100 mM NaCl, 0.01% NP-40, 10% glycerol, and 1 mM DTT). Proteins were then eluted on ice with buffer B supplemented with 0.5 mg/ml 3× HA peptide (I2149, Sigma).

### *In vitro* ubiquitination assays

*In vitro* ubiquitination assays were performed using a ubiquitination kit (BML-UW9920, Enzo), according to the manufacturer’s instructions. Briefly, reactions were assembled in 1.5-ml tubes in a final volume of 50 μl containing 14 μl of dH_2_O, 5 μl of 10× ubiquitinylation buffer, 2.5 μl of 20× ubiquitin, 2.5 μl of 20× E1 enzyme, 5 μl of 10× E2 conjugating enzyme, 5 μg of SCF^FBXO22^ E3 complex, and 5 μg of recombinant HDAC1, RCOR1, or LSD1 protein (custom-made, MedChemExpress). The reaction was initiated by adding 2.5 μl of Mg-ATP solution, gently mixed, and incubated at 37°C for 3 h. The reaction was terminated by adding 1% SDS, followed by dilution with 300 μl of denaturing IP buffer. The diluted samples were then incubated with Dynabeads (10003D, ThermoFisher) pre-bound to anti-HDAC1, anti-RCOR1, or anti-LSD1 antibodies on a rotary shaker at 4°C for 4 h. After washing three times with denaturing IP buffer, bound proteins were eluted in 2% SDS loading buffer and subjected to western blot analysis.

### RNA extraction and quantitative real-time PCR (RT-PCR)

Total RNA was extracted from cells and tissues using TRIzol reagent (AG21101, Accurate Biology), according to the manufacturer’s instructions. cDNA synthesis was performed using the Evo M-MLV RT Kit with gDNA Eraser (AG11705, Accurate Biology). RT-PCR analysis employed SYBR Green chemistry with the following cycling protocol: initial denaturation at 95°C for 30 s, followed by 40 cycles of denaturation at 95°C for 5 s, annealing and extension at 61°C for 30 s, and finally a last melt curve step of 95°C for 15 s, 60°C for 1 min, and 95°C for 1s. Gene expression was quantified using the comparative ΔΔCt method. *GAPDH* or *H2A* were used as reference genes. The primer sequences used are provided in Supplementary Table S3.

### RNA-seq and data analysis

Total RNA was extracted from control and *FBXO22* knockdown hTSCs, as well as from chorionic villi of normal pregnancies and RPL patients, using the previously described TRIzol method [34]. RNA quality and integrity were assessed using an Agilent 2100 Bioanalyzer. Quality compliant RNA samples were sent to Novogene for library construction and sequencing. Sequencing was performed on the Illumina NovaSeq 6000 platform with three biological replicates per group. Clean reads were aligned to the human reference genome (GRCh38) using STAR, and differentially expressed genes were identified using DESeq2.

### Histological and immunostaining analysis

Human villous tissues were fixed in 4% paraformaldehyde, routinely dehydrated through ethanol and xylene, and embedded in paraffin. Sections (5 μm thickness) were deparaffinized in xylene and ethanol, subjected to antigen retrieval, peroxidase inactivation, and blocked with 3% BSA. Subsequently, sections were incubated overnight at 4°C with primary antibodies. After incubation with corresponding HRP-conjugated secondary antibodies, staining was developed with DAB chromogen, followed by hematoxylin counterstaining and mounting. For H&E staining, deparaffinized sections were stained with hematoxylin and eosin prior to mounting and examination. The following primary antibodies were used: anti-FBXO22 (1:1000, Proteintech), anti-HDAC1 (1:2000, CST), anti-RCOR1 (1:1000, ABclonal), anti-LSD1 (1:2000, ABclonal), anti-CDH1 (1:2000, CST), anti-TEAD4 (1:1000, GeneTex), anti-CGB (1:2000, ThermoFisher), anti-SDC1 (1:3000, Abcam), and anti-CK7 (1:3000, Abcam). Whole-slide images were acquired using an Olympus VS200 slide scanner.

### Flow cytometry

Cell cycle analysis was performed by flow cytometry using the cell cycle assay kit (E-CK-A351, Elabscience) according to the manufacturer’s instructions. In brief, approximately 5 × 10^5^ cells were collected, washed with PBS, and fixed in pre-chilled 100% ethanol at −20°C overnight. After washing, cells were resuspended in PBS and incubated at room temperature for 15 min. RNase A was added and incubated at 37°C for 30 min, followed by staining with propidium iodide for 30 min in the dark. Samples were analyzed by flow cytometry with 488 nm excitation to assess cell cycle distribution.

### Cell proliferation assay

hTSCs were seeded into 96-well microtiter plates at a density of approximately 1500 cells per well. Cell proliferation was evaluated at 0, 24, 48, and 72 h using the CCK-8 assay (HY-K0301, MedChemExpress). At each time point, 10 μl of cell proliferation assay (CCK-8) reagent was added to each well and incubated at 37°C for 2 h. The absorbance at 450 nm was then measured with a microplate reader to determine cell viability.

### EdU incorporation assay

Cell proliferation was evaluated using an EdU Cell Proliferation Kit (E-CK-A376, Elabscience Biotechnology). Briefly, cells were incubated with 10 μM EdU for 1 h, fixed with 4% paraformaldehyde, and permeabilized with 0.3% Triton X-100. Cells were then incubated with Click reaction solution, followed by 4′,6-diamidino-2-phenylindole (DAPI) staining. Images were captured under microscope.

### Immunofluorescence assay

Cells cultured in confocal dishes were fixed with 4% paraformaldehyde at room temperature, permeabilized with 0.1% Triton X-100, and blocked with 3% BSA. Samples were incubated with primary antibodies overnight at 4°C. The following primary antibodies were used: anti-FBXO22 (1:1000, Proteintech), anti-HDAC1 (1:1000, CST), anti-LSD1 (1:1000, ABclonal), anti-CDH1 (1:2000, CST), anti-CGB (1:2000, ThermoFisher), anti-GATA3 (1:2000, CST), anti-TP63 (1:2000, CST), and anti-HLA-G (1:1000, Proteintech). After incubation with Cy2, Cy3, or Cy5-conjugated secondary antibodies (1:1000, Abcam) for 1 h at room temperature, nuclei were counterstained with DAPI (1 μg/ml). Human villous tissues and trophoblast organoids were processed identically using 5 μm paraffin sections. Images were captured with a Nikon AX confocal system and processed using NIS-Elements Viewer software.

### Proximity ligation assay

Proximity ligation analysis was performed using the Duolink® In Situ Red Starter Kit (DUO92008, ThermoFisher), according to the manufacturer’s instructions. Briefly, cells on coverslips from control and *FBXO22* knockdown groups were fixed with 4% paraformaldehyde for 10 min, washed three times with PBS, and permeabilized with 0.5% Triton X-100 for 10 min, followed by another PBS wash. Coverslips were blocked with 5% BSA for 30 min and incubated overnight at 4 °C with appropriately diluted primary antibodies. Chorionic villus tissue sections were similarly prepared and incubated with primary antibodies at 4°C overnight. The next day, after PBS washes, PLA probes (PLUS and MINUS, 19 μl each) were applied and incubated at 37°C for 1 h. Following probe washes, ligation was carried out at 37°C for 30 min, and amplification was performed at 37°C for 100 min. Coverslips were sequentially washed with wash buffer, counterstained with DAPI, mounted, and imaged using a confocal microscope.

### Engraftment of hTSCs into NOD-SCID mice

All mice were housed in the animal care facility of Chongqing Medical University according to the guidelines for the care and use of laboratory animals. All animal experiments were approved by the Medical Ethics Committee of Chongqing Medical University (No. 2022185). hTSC transplantation into NOD-SCID mice was performed following previously described protocols [10, 13]. Briefly, hTSCs were cultured in hTSC medium for 3 days and then induced in STB differentiation medium for 1 day. Once the cell density reached ~80% (~5 × 10⁶ cells), the cells were dissociated using TrypLE Express. The harvested cells were resuspended in 120 μl of a 1:2 mixture of Matrigel and DMEM/F12 containing 0.3% BSA and 1% ITS-X supplement. The cell suspension was subcutaneously injected into the dorsal region of 6–8-week-old male NOD-SCID mice (purchased from GemPharmatech). On day 7 post-transplantation, grafts were collected, fixed overnight in 4% paraformaldehyde at 4°C, dehydrated through an ethanol gradient, and embedded in paraffin. Tissue sections were processed for H&E staining, immunohistochemistry, and immunofluorescence according to standard protocols. Blood was collected from mice, and the serum was separated (4°C, 3000 rpm, 15 min) and stored at −80°C.

### Measurement of serum human chorionic gonadotropin

The human chorionic gonadotropin (hCG) levels in mouse serum samples were quantified using a sandwich ELISA (Elabscience) following the manufacturer’s protocol. In brief, standards and samples were added to antibody-precoated wells and incubated at 37°C for 90 min. After washing, biotinylated detection antibodies and HRP-conjugated reagents were added sequentially and incubated for 1 h and for 30 min, respectively, with washing between each step. Color development was achieved by adding TMB substrate and incubating in the dark for approximately 15 min, after which the reaction was terminated with a stop solution. The absorbance was immediately read at 450 nm using a microplate reader, and hCG concentrations were determined from a standard calibration curve.

### Protein structure prediction, docking, and molecular dynamics simulation

To predict and analyze the interaction between CoREST and FBXO22, we first constructed the full-length structure of CoREST using AlphaFold3 [36]. The amino acid sequence of CoREST was retrieved from the UniProt database and used as the input for structure prediction. The interaction between CoREST and FBXO22 (AlphaFold-predicted geometry) was then examined through docking simulations using HDOCK [37], and the docking results were further visualized and analyzed with PyMOL. To explore the structural stability and dynamic behavior of the complex, molecular dynamics (MD) simulations were performed using the GROMACS-2022 software package based on the docking geometry [38]. The protein was parameterized with the Amber ff99SB force field, and water molecules were represented by the TIP3P model [39]. The system was first subjected to energy minimization (50 000 steps, steepest descent method), followed by equilibration in the NVT and NPT ensembles for 100 ps each, with positional restraints on the heavy atoms. Subsequently, a 100-ns production simulation was carried out under constant temperature (310 K) and pressure (1 bar), maintained by the Parrinello–Rahman method. Long-range electrostatic interactions were computed using the Particle-Mesh-Ewald (PME) method [40], with a 1 nm cutoff for van der Waals interactions, and all bonds involving hydrogen were constrained using the SHAKE algorithm. Trajectories were saved every 10 ps, and the binding free energies of the complexes were finally estimated using the molecular mechanics Poisson–Boltzmann surface area (MM/PBSA) method based on the MD simulations [41–43].

### ChIP-seq and ChIP-qPCR

Chromatin immunoprecipitation followed by sequencing (ChIP-seq) was performed as described previously [31]. In brief, hTSCs were crosslinked in 1% formaldehyde (2606, CST) at room temperature for 10 min, followed by quenching the crosslinking reaction with 0.125 M glycine. After cell collection, nuclear lysis, precipitation, and resuspension were performed sequentially using Lysis Buffer 1 (50 mM HEPES pH 7.5, 1 mM EDTA pH 8.0, 140 mM NaCl, 0.5% NP-40, 10% glycerol, and 0.25% Triton X-100), Lysis Buffer 2 (10 mM Tris–HCl pH 8.0, 1 mM EDTA pH 8.0, 0.5 mM EGTA pH 8.0, and 200 mM NaCl), and Lysis Buffer 3 (10 mM Tris–HCl pH 8.0, 1 mM EDTA pH 8.0, 0.5 mM EGTA pH 8.0, 100 mM NaCl, 0.1% sodium deoxycholate, and 0.1% *N*-laurylsarcosine sodium) with proteinase inhibitor cocktail (Roche, 04693132001) added to all buffers before use. Chromatin was sonicated at 4°C using a sonicator (Qsonica) with the following parameters: 14 cycles of 30 s on/30 s off, to fragment the chromatin to 300–500 bp. A portion (5%) of the sonicated chromatin was saved as input control, while the remaining chromatin was incubated overnight at 4°C with H3K27ac antibody (CST, 4658) for immunoprecipitation. Then, 30 μl of Protein A/G magnetic beads (88803, ThermoFisher) were added, and incubation continued for 3 h at 4°C. After washing and elution, the protein–DNA crosslinks were reversed by incubation at 65°C overnight. Immunoprecipitated DNA was purified using a PCR Purification Kit (QIAGEN, 28106) and quantified using a Qubit 4.0. The purified DNA was used to construct sequencing libraries using the VAHTS Universal DNA Library Prep Kit (Vazyme, ND610) according to the manufacturer’s instructions and sequenced on the Illumina NovaSeq 6000 platform. The raw sequencing data were processed using the Illumina Genome Analysis pipeline, aligned to the human reference genome hg38, and ChIP-seq tracks were visualized using IGV software (v2.12.3).

Purified DNA was subjected to qPCR analysis. Input was included in each reaction, and data from control and *FBXO22* knockdown hTSCs were normalized to the Input. Specific primers targeting the regulatory regions of the genes, which were designed based on the ChIP-seq and CUT&Tag signal distribution, were used for detection, and the primer sequences used are provided in Supplementary Table S3. Relative enrichment was calculated based on the comparative Ct method to assess the binding levels at the target regions.

### CUT&Tag assay

The CUT&Tag assay was performed using the NovoNGS CUT&Tag 4.0 High-Sensitivity Kit (Novoprotein) according to the manufacturer’s instructions. Briefly, hTSCs were collected and washed with Wash Buffer. Cells were then bound to ConA magnetic beads for 10 min at room temperature. Then, cells were incubated with H3K9me2 antibody (39041, Active Motif) overnight at 4°C. Following primary antibody binding, samples were washed and incubated with goat anti-rabbit IgG secondary antibody for 1 h. Tagmentation was carried out using the ChiTag pAG transposome for 1 h at room temperature. The reaction was terminated with Stop Buffer and Proteinase K, and DNA fragments were purified using Tagment DNA Extract Beads. Libraries were amplified via PCR with index primers and purified with DNA Clean Beads. Sequencing was performed on the Illumina NovaSeq 6000 platform. The raw sequencing data were processed using the Illumina Genome Analysis pipeline, aligned to the human reference genome hg38, and CUT&Tag tracks were visualized using IGV software (v2.12.3).

### Luciferase reporter assay

DNA sequences containing the promoter regions of *CTSD, ERVFRD-1, GCM1, CGB7, SDC1, GATA3*, and *TEAD4* from human genomic DNA were cloned into the firefly luciferase reporter vector pGL3. Twenty-four h after *FBXO22* knockdown, the firefly luciferase reporter vector pGL3 together with the renilla luciferase control reporter vector pRL-TK were used to transfect HEK293T cells using Lipofectamine 3000 (Invitrogen). Cells were collected 48 h post-transfection, and luciferase activities were determined using the Dual-Luciferase Reporter Assay System (Promega, E1910).

### Statistical analysis

Statistical analyses were performed using GraphPad Prism v9.0 (GraphPad Software, USA). At least three independent samples were included in each experiment, data are presented as mean ± standard error of the mean (SEM). Before applying parametric tests, normality was assessed using the Shapiro–Wilk test to ensure that the data satisfied the assumption of normal distribution. For comparisons between two groups, unpaired two-tailed *t* tests were used; for comparisons among three or more groups, one-way analysis of variance (one-way ANOVA) followed by Tukey’s multiple comparisons test was applied. A *P*-value of <0.05 was considered statistically significant. *P < 0.05, ** *P* < 0.01, *** *P* < 0.001, **** *P* < 0.0001. *q* < 0.05 was considered significant for GSEA.

## Results

### FBXO22 is localized to the nuclei of CTBs

**Figure 1.**
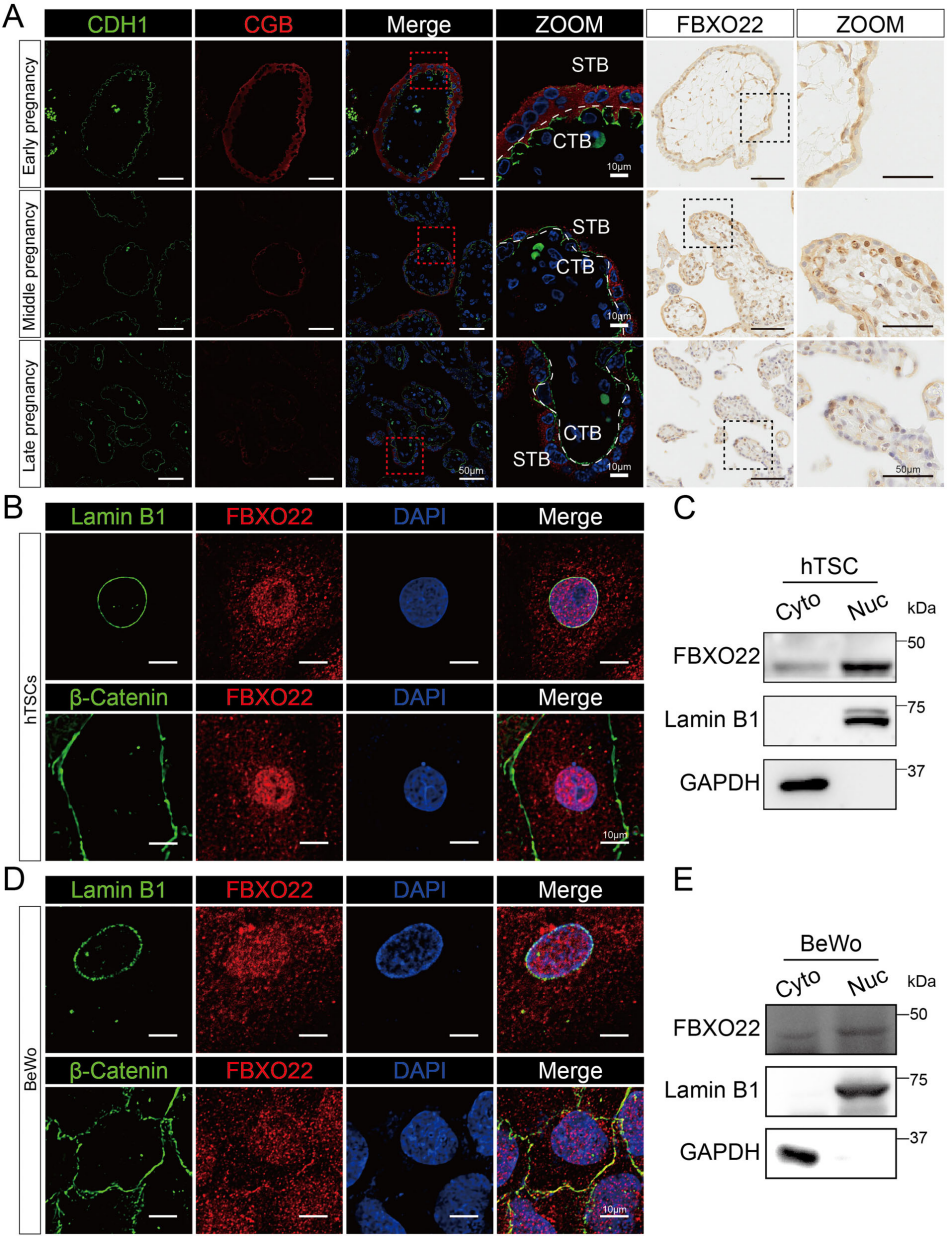
FBXO22 is a nuclear-localized E3 ligase in trophoblasts. (**A**) Immunostaining of FBXO22 in first-, second-, and third-trimester placental villi. CDH1 and CGB were used as markers for cytotrophoblasts (CTBs) and syncytiotrophoblasts (STBs), respectively. DAPI stained the nuclei. (**B**) Immunofluorescence analysis of FBXO22 in hTSCs. β-Catenin and LaminB1 were used as markers for the plasma membrane and nuclear envelope, respectively. (**C**) Western blot analysis of FBXO22 in cytoplasmic and nuclear fractions of hTSCs. GAPDH and LaminB1 were used as markers for the cytoplasm and nucleus, respectively. Cyto: cytoplasm; Nuc: nucleus. (**D**) Immunofluorescence analysis of FBXO22 in BeWo cells. (**E**) Western blot analysis of FBXO22 in cytoplasmic and nuclear fractions of BeWo cells. The data presented are representative of at least three independent experiments.

To investigate the role of FBXO22 in placental trophoblast development, we first examined its expression in human placental villi from the first and second trimesters, as well as in term placenta. Immunostaining revealed that FBXO22 was predominantly localized to the nuclei of CDH1-positive CTBs, a pattern distinct from that in CGB-positive STBs (Fig. 1A). To further elucidate the location of FBXO22 in human trophoblast cells, we utilized hTSCs derived from first-trimester CTBs, which expressed canonical stem cell markers (TP63, GATA3, and CDH1) and retained the potential to differentiate into both STBs (CGB-positive) and EVTs (HLA-G-positive) (Supplementary Fig. S1A–C). In addition, we also established human TOs derived from hTSCs, which self-organized into spherical structures consisting of an outer layer of CTBs stably expressing CDH1 and an inner compartment containing spontaneously differentiated CGB-positive STBs (Supplementary Fig. S1C). Consistent with our tissue analysis, FBXO22 was predominantly localized to the nuclei of hTSCs and the choriocarcinoma cell line BeWo cells (Fig. 1B–E). Collectively, these findings indicate that FBXO22 is a nuclear-enriched ubiquitin ligase in CTBs, suggesting a potential role in trophoblast fate decisions during early placental development.

### FBXO22 deficiency promotes premature trophoblast syncytialization

**Figure 2.**
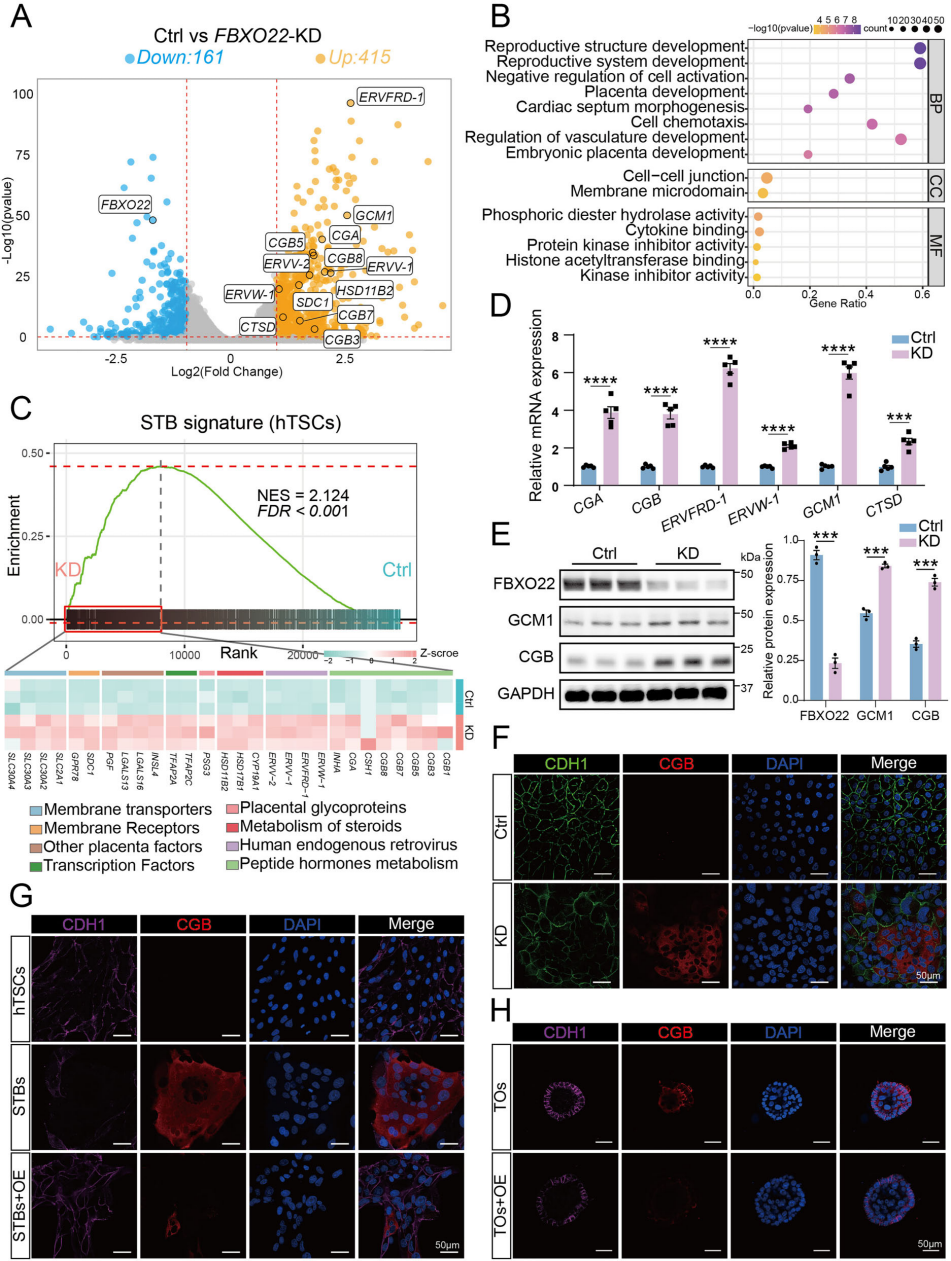
FBXO22 deficiency facilitates aberrant trophoblast syncytialization. (**A**) Volcano plot of genes differentially expressed in siRNA mediated *FBXO22* knockdown (*FBXO22*-KD) and control hTSCs [*P* < 0.05, log_2_ (fold change) > 1]. (**B**) Gene ontology (GO) enrichment analysis of genes downregulated in *FBXO22*-KD hTSCs. BP, biological process; CC: cellular component; MF: molecular function. (**C**) Gene set enrichment analysis (GSEA) of STB signature in *FBXO22*-KD and control hTSCs. (**D**) RT-PCR analysis of STB marker expression in *FBXO22*-KD and control hTSCs. The values were normalized to *GAPDH; n* = 5. (**E**) Western blot analysis of STB marker expression in *FBXO22*-KD and control hTSCs. GAPDH was used as the loading control; *n* = 3. (**F–H**) Immunofluorescent staining of CDH1 and CGB in (F) *FBXO22*-KD and control hTSCs; (G) STBs, STBs + overexpressed *FBXO22* (OE-*FBXO22*) and control hTSCs; (H) OE-*FBXO22* and control TOs. DAPI stains the nuclei. The data presented are representative of at least three independent experiments (F–H). Data are means ± SEM; ***P < 0.001, ****P < 0.0001 by unpaired two-tailed *t* test.

Building on a previous report of reduced *FBXO22* expression in villous tissues from RPL patients [28], we investigated the functional consequences of its depletion. We performed whole-transcriptome RNA-seq of hTSCs following siRNA-mediated knockdown of *FBXO22* and observed 161 down-regulated and 415 up-regulated genes (Fig. 2A and Supplementary Dataset S1). Genes associated with syncytialization, including *CGB3, CGB5, CGB7, CGB8, CGA, SDC1, GCM1, ERVW-1, ERVFRD-1, ERVV-1, ERVV-2, CTSD*, and *HSD11B2* (Fig. 2A and Supplementary Fig. S2A) were significantly upregulated in *FBXO22*-depleted cells. Moreover, gene ontology (GO) analysis confirmed the enrichment of pathways related to reproductive structure morphogenesis, placental development, and cell junction organization, all of which are essential for trophoblast fusion (Fig. 2B). To systematically evaluate the impact of FBXO22 expression on STB lineage specification, we defined an “STB signature” gene set based on previously reported STB transcriptomes (GSE209977) and performed gene set enrichment analysis (GSEA) (Fig. 2C) [12, 44]. We found a pronounced transcriptional shift toward the STB lineage in *FBXO22*-deficient hTSCs. This included genes involved in hormone metabolism (the *CGB* family, *INHA*), steroidogenic enzymes (*CYP19A1, HSD17B1, HSD11B2*), transcription factors associated with early trophoblast progenitor specification (*TFAP2A, TFAP2C*), placental glycoproteins (*PSG3*), fusogenic proteins (*ERVW-1, ERVV-1/2, ERVFRD-1*), membrane transporters (*SLC2A1, SLC30* family), and placenta specific factors and receptors (*INSL4, LGALS13/16, PGF, SDC1, GPR78*). Consistently, western blot, RT-PCR, immunofluorescence, and bright-field images collectively showed that *FBXO22* depletion markedly promoted STB differentiation, rather than EVT differentiation (Fig. 2D–F, Supplementary Figs S2C and S3). This enhanced differentiation was accompanied by impaired proliferation and cell cycle arrest in the G2/M phase (Supplementary Fig. S2D–F). Interestingly, we observed that FBXO22 was decreased at 72 h of STB induction (Supplementary Fig. S2G). Based on this observation, we conducted reverse validation through *FBXO22* overexpression. Strikingly, overexpression of *FBXO22* significantly suppressed the expression of STB markers (Fig. 2G and H, and Supplementary Fig. S2H) and inhibited FSK-induced cell fusion in BeWo cells (Supplementary Fig. S2I). Together, these knockdown and overexpression experiments demonstrate that FBXO22 predominantly localizes to hTSCs nuclei and functions as a negative regulator of trophoblast syncytialization.

### FBXO22 directly interacts with, and regulates, the CoREST complex

**Figure 3.**
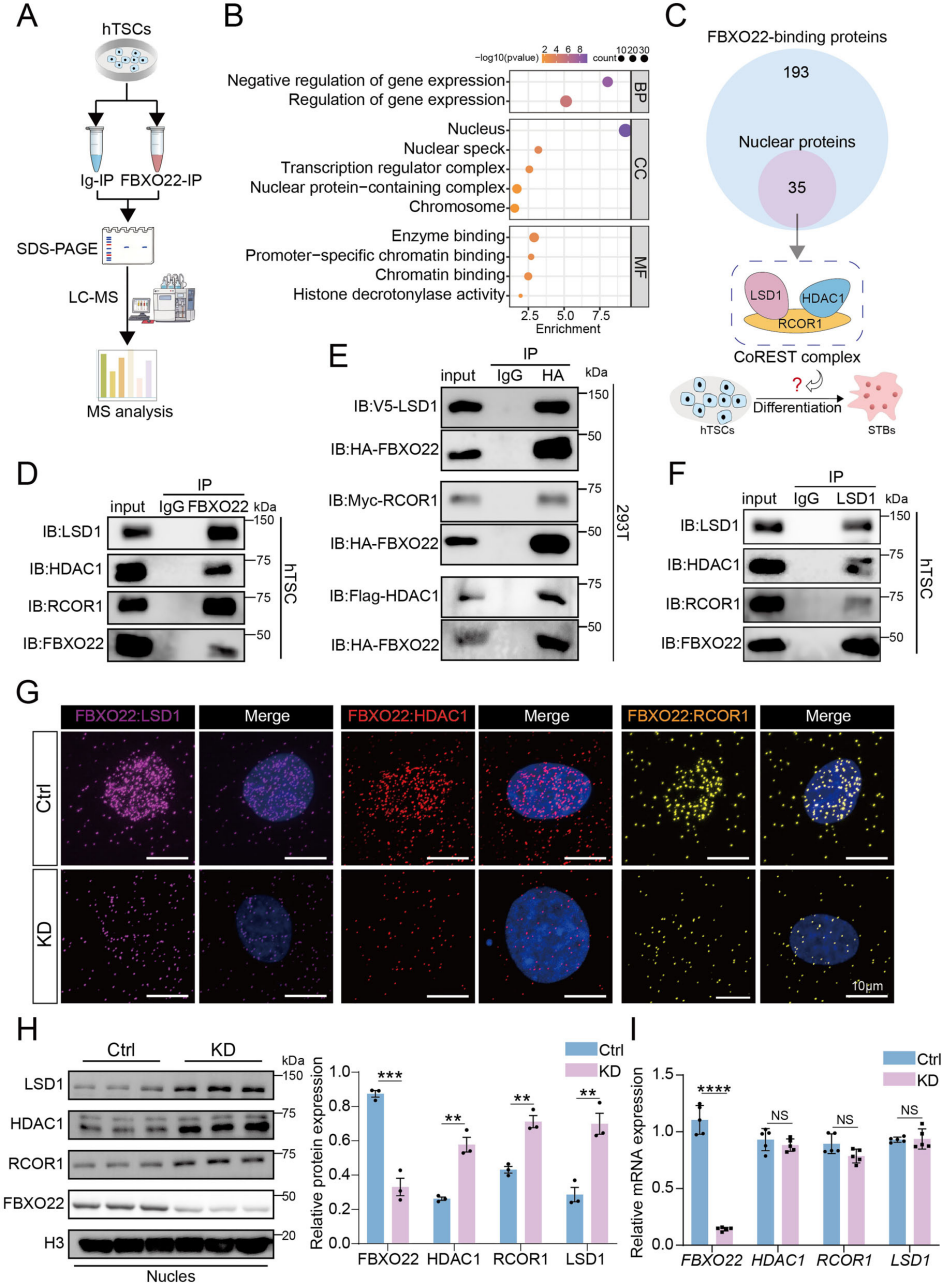
FBXO22 interacts directly with the CoREST complex. (**A**) Schematic diagram showing the workflow for the identification of FBXO22 interactors. (**B**) GO enrichment analysis of FBXO22-interacting nuclear proteins in hTSCs. (**C**) Venn diagram showing the nuclear proteins among the FBXO22 interactors. (**D**) Co-IP analysis of endogenous interactions between FBXO22 and the CoREST complex in hTSCs. (**E**) Co-IP analysis of exogenous interactions between FBXO22 and the CoREST complex in HEK293T cells (293T). (**F**) Co-IP analysis of endogenous interactions between LSD1, HDAC1, RCOR1, and FBXO22 in hTSCs. (**G**) PLA showing the proximity between FBXO22 and CoREST in *FBXO22*-KD cells and control hTSCs. (**H**) Western blot analysis of CoREST complex proteins in nuclear extracts from control and *FBXO22*-KD hTSCs. H3 was used as the nuclear loading control; *n* = 3. (**I**) RT-PCR analysis of CoREST complex expression in *FBXO22*-KD cells and control hTSCs. The values were normalized to *GAPDH; n* = 5. NS, no significance. Data are means ± SEM, and are representative of at least three independent experiments (D–G); **P < 0.01, ***P < 0.001, ****P < 0.0001 by unpaired two-tailed *t* test. The Eppendorf tube images in Fig. 3A were adapted from SciDraw under the Creative Commons license CC BY 4.0.

To elucidate the molecular mechanism of FBXO22 function, we performed co-immunoprecipitation (Co-IP) combined with mass spectrometry (Co-IP/MS) in hTSCs, and identified 193 candidate interacting proteins (Fig. 3A and Supplementary Dataset S3). Considering the nuclear localization of FBXO22 in hTSCs, we focused on the 35 nuclear candidates. GO enrichment analysis revealed significant enrichment for negative transcriptional regulation, chromatin binding, and histone modification (Fig. 3B). These results suggest that FBXO22 may be integrated into transcriptional regulatory complexes, interact with chromatin, and recognize promoter regions, influence histone modifications, and thereby regulate gene expression (Fig. 3B). Among these interacting proteins, we prioritized HDAC1, LSD1, and RCOR1 (Fig. 3C), which together form the CoREST transcriptional repressor complex, due to their established roles in trophoblast differentiation [17, 19]. Endogenous Co-IP assays in hTSCs confirmed a robust interaction between FBXO22 and the CoREST complex (Fig. 3D), which was further validated by exogenous Co-IP in HEK293T cells (Fig. 3E). Consistently, immunofluorescence analysis demonstrated prominent nuclear co-localization of FBXO22 with CoREST components (Supplementary Fig. S4A and B), while the proximity ligation assay (PLA) also provided direct evidence of close spatial proximity in the nucleus between FBXO22 and CoREST components (Fig. 3G and Supplementary Fig. S4C).

To investigate the binding mode of FBXO22 with the CoREST complex, we performed molecular dynamics simulations. The binding complex exhibited minimal overall root-mean-square deviation (RMSD) fluctuations, indicating a stable interaction (Supplementary Fig. S5A). The root-mean-square fluctuation (RMSF) analysis revealed that certain regions of CoREST were highly flexible, likely facilitating conformational adjustments at the binding interface (Supplementary Fig. S5B). The radius of gyration (*R*_g_) fluctuated by only 0.2 nm, with the binding complex maintaining a compact overall structure without signs of loosening or aggregation (Supplementary Fig. S5C). Free energy landscape analysis further confirmed that the system remained in a stable low-energy state throughout the simulation (Supplementary Fig. S5D). FBXO22 remained consistently associated with the surface of CoREST, demonstrating strong dynamic stability (Supplementary Fig. S5E and F). Molecular mechanics-generalized-Born surface area (MMGBSA) binding energy calculations showed that the total binding free energy of the FBXO22-CoREST complex was Δ*E*tot = −172.41 kcal/mol, indicating a spontaneous and thermodynamically favorable interaction (Supplementary Fig. S5G). Per-residue energy decomposition identified key residues mediating the interaction, with LSD1 in the CoREST complex contributing most significantly to the interaction (Supplementary Fig. S5H). These results indicate a thermodynamically stable and specific interaction between FBXO22 and the CoREST complex.

To independently validate the interaction between the CoREST complex and FBXO22, we selected LSD1 as a representative component of the CoREST complex, and endogenous Co-IP verified that LSD1, HDAC1, RCOR1, and FBXO22 interact in hTSCs (Fig. 3F). Finally, to capture the broader interaction network of LSD1, we used TurboID labeling. TurboID is a promiscuous biotin ligase that, when fused to a protein of interest, biotinylates nearby proteins within living cells, enabling highly sensitive mapping of protein–protein interaction networks under native conditions [45]. We expressed LSD1-TurboID in HEK293T cells and performed biotin-based proximity labeling followed by MS analysis. We identified 2 389 potential LSD1 interactors (Supplementary Fig. S6A and B). Comparison with a list of human ubiquitin ligases, 25 potential E3 ligases were identified, among which FBXO22 was a top candidate. Crucially, knockdown of *FBXO22* in hTSCs led to a marked accumulation of CoREST complex proteins without altering their mRNA levels significantly (Fig. 3H–I), indicating that FBXO22 regulates the complex post-translationally. Together, these results demonstrate that FBXO22 directly interacts with and modulates the stability of CoREST complex in hTSCs.

### FBXO22 mediates proteasomal degradation of the CoREST complex and restricts syncytialization

**Figure 4.**
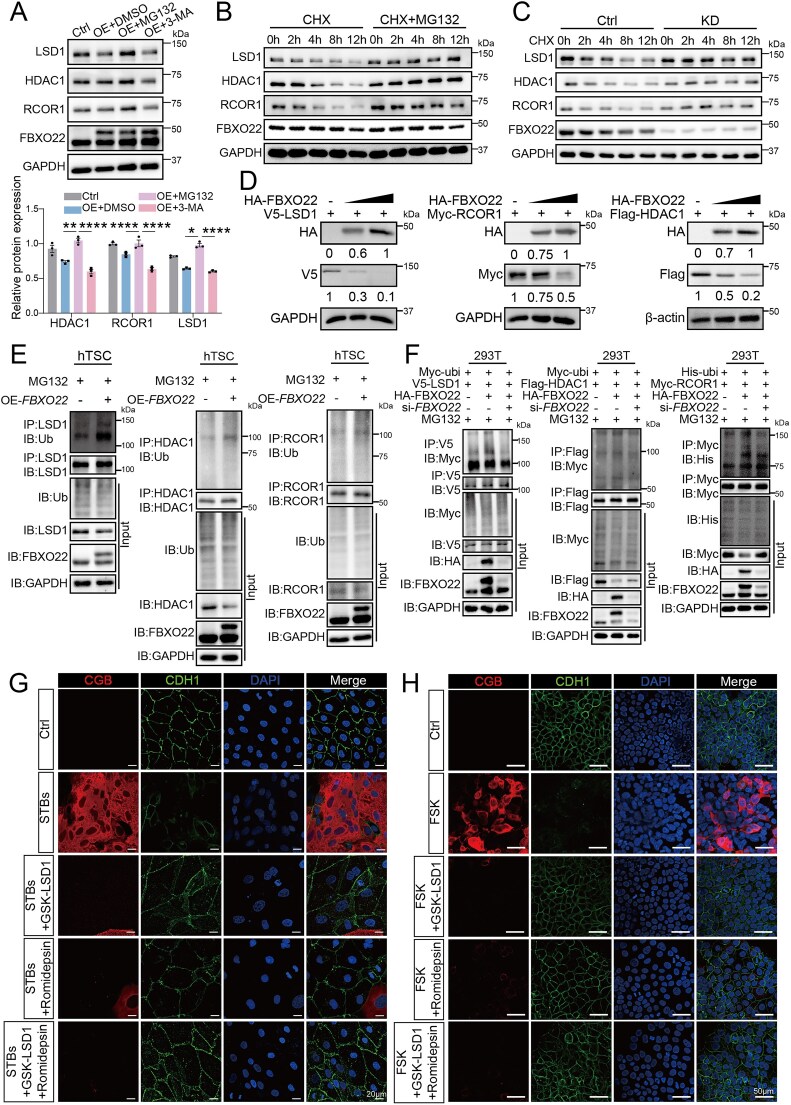
FBXO22-mediated ubiquitination of the CoREST complex orchestrates trophoblast syncytialization. (**A**) Western blot analysis of CoREST complex proteins and FBXO22 in *FBXO22*-OE cells and control hTSCs treated with MG132 or 3-MA for 6 h. (**B**) Western blot analysis of FBXO22 and CoREST complex protein levels in hTSCs treated with cycloheximide (CHX) or CHX combined with MG132 for 0, 2, 4, 8, and 12 h. (**C**) Western blot analysis of FBXO22 and CoREST complex protein levels in *FBXO22*-KD cells and control hTSCs following CHX treatment for 0, 2, 4, 8, and 12 h. (**D**) Western blot analysis of V5-LSD1, Myc-RCOR1, and Flag-HDAC1 protein levels with increasing doses of HA-FBXO22 expression plasmid in HEK293T cells. (**E**) Western blot analysis of CoREST complex ubiquitination in *FBXO22*-OE cells and control hTSCs. (**F**) Western blot analysis of CoREST complex ubiquitination in HEK293T cells co-transfected with an FBXO22 expression plasmid and si-*FBXO22*. (**G**) Immunofluorescence staining of CDH1 and CGB in hTSCs, hTSCs-derived STBs, and hTSCs-derived STBs treated with Romidepsin (2 nM) or GSK-LSD1 (1 μM) for 72 h. (**H**) Immunofluorescence staining of CDH1 and CGB in BeWo cells treated with DMSO, Forskolin (FSK), or FSK combined with Romidepsin (2 nM) or GSK-LSD1 (1 μM) for 48 h. Data are means ± SEM; GAPDH or β-actin was used as the loading control; the data presented are representative of at least three independent experiments; *P < 0.05, **P < 0.01, ***P < 0.001, ****P < 0.0001 by unpaired two-tailed *t* test and one-way ANOVA.

We next sought to confirm that FBXO22, as an E3 ligase, mediates the degradation of the CoREST complex. Overexpression of *FBXO22* markedly reduced the abundance of CoREST proteins. This reduction was prevented by the proteasome inhibitor MG132, but not by the autophagy inhibitor 3-MA (Fig. 4A). In cycloheximide (CHX) chase assays, knockdown of *FBXO22* or treatment with MG132 substantially stabilized CoREST proteins, extending their half-life (Fig. 4B and C, and Supplementary Fig. S7A and B). Conversely, titrating FBXO22 expression in HEK293T cells resulted in a dose-dependent decrease in CoREST protein levels (Fig. 4D). Together, these data establish that FBXO22 destabilizes the CoREST complex via proteasome-dependent degradation.

To determine whether FBXO22 directly promotes CoREST ubiquitination, we performed endogenous ubiquitination assays. We found that FBXO22 overexpression markedly enhanced CoREST polyubiquitination (Fig. 4E). Exogenous ubiquitination assays demonstrated that FBXO22 knockdown significantly attenuated CoREST ubiquitination (Fig. 4F). To further define the ubiquitin linkage type involved, we performed *in vivo* ubiquitination assays using K48R and K63R ubiquitin mutants. FBXO22-induced ubiquitination of LSD1, HDAC1, and RCOR1 was markedly reduced in the presence of the K48R mutant, whereas the K63R mutant had little effect, indicating that FBXO22 primarily mediates K48-linked polyubiquitination of CoREST complex components (Supplementary Fig. S7C). Importantly, an *in vitro* ubiquitination assay using immunopurified SCF^FBXO22^ complex together with recombinant substrate proteins further demonstrated direct FBXO22-dependent ubiquitination of LSD1, HDAC1, and RCOR1 (Supplementary Fig. S7D and E). These findings establish the CoREST complex as a bona fide substrate of the SCF^FBXO22^ E3 ligase. We next investigated the functional consequences of FBXO22-mediated CoREST degradation in trophoblast differentiation. Pharmacological inhibition of HDAC1 (Romidepsin) or LSD1 (GSK-LSD1) impaired STB formation (Fig. 4G), as evidenced by reduced CGB expression and diminished formation of multinucleated cell clusters in FSK-induced BeWo cell fusion (Fig. 4H). Consistent with these results, inhibition of HDAC1 and LSD1 led to a significant decrease in the mRNA expression of STB marker genes (*CGA, CGB, ERVFRD-1, GCM1, ERVW-1*, and *CTSD*) (Supplementary Fig. S8A), while western blot confirmed decreased GCM1 expression and partial restoration of the stemness factor TEAD4 (Supplementary Fig. S8B). Collectively, these results demonstrate that FBXO22 restricts trophoblast syncytialization by catalyzing CoREST transcriptional repressor complex polyubiquitination and promoting its proteasomal degradation.

### FBXO22 deficiency promotes aberrant STB formation by altering H3K27ac and H3K9me2 landscapes

**Figure 5.**
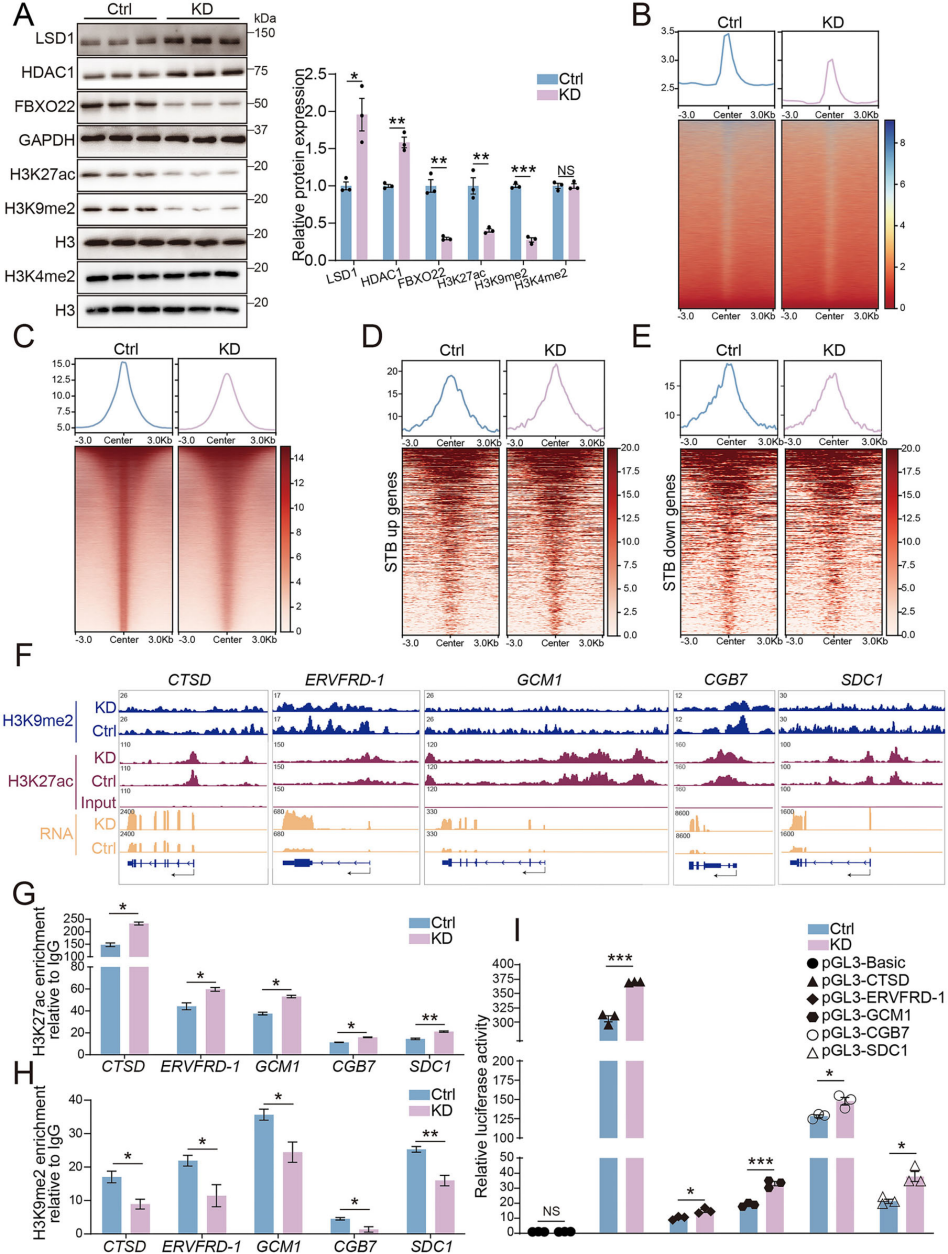
FBXO22 deficiency facilitates aberrant syncytialization via coordinated histone acetylation and methylation. (**A**) Western blot analysis and quantification of FBXO22, CoREST complex, H3K27ac, and H3K9me2 protein levels in *FBXO22*-KD cells and control hTSCs. GAPDH and H3 were used as the loading controls; *n* = 3. H3K27ac: histone 3 lysine 27 acetylation; H3K9me2: histone 3 lysine 9 dimethylation. NS, no significance. (**B**) Line plots and heatmap showing H3K9me2 CUT&Tag peaks in *FBXO22*-KD cells and control hTSCs. (**C**) Line plots and heatmap showing H3K27ac ChIP-seq peaks in *FBXO22*-KD cells and control hTSCs. (**D**) Line plots and heatmap showing H3K27ac ChIP-seq peaks of STB-upregulated genes in *FBXO22*-KD cells and control hTSCs. (**E**) Line plots and heatmap showing H3K27ac ChIP-seq peaks of STB-downregulated genes in *FBXO22*-KD cells and control hTSCs. (**F**) Genome browser view of normalized RNA-seq tracks and ChIP-seq signals of H3K27ac, and CUT&Tag signals of H3K9me2 at the STB marker gene loci. (**G**) ChIP-qPCR of H3K27ac modifications in the promoter regions of STB marker genes in *FBXO22*-KD cells and control hTSCs; *n* = 3. (**H**) ChIP-qPCR of H3K9me2 modifications in the promoter regions of STB marker genes in *FBXO22*-KD cells and control hTSCs; *n* = 3. (**I**) Luciferase reporter assays of the activities of STB marker gene promoters in *FBXO22*-KD and control cells; *n* = 3. NS, no significance. Data are means ± SEM; *P < 0.05, **P < 0.01 and ***P < 0.001 by unpaired two-tailed *t* test (A, G–I).

The CoREST complex functions as a transcriptional repressor through HDAC1-mediated deacetylation (e.g. H3K27ac) and LSD1-mediated demethylation (e.g. H3K4me1/2 and H3K9me2). Since FBXO22 depletion led to CoREST complex accumulation, we examined three histone modifications, including H3K27ac, H3K9me2, and H3K4me2 modifications. Intriguingly, in *FBXO22*-KD hTSCs, elevated HDAC1 and LSD1 protein levels were accompanied by global reductions in both H3K27ac and H3K9me2, whereas H3K4me2 remained largely unaffected (Fig. 5A). Notably, during STB formation, global H3K27ac declined whereas H3K9me2 remained largely unchanged. Conversely, *FBXO22* overexpression reduced both histone marks (Supplementary Fig. S9A). Thus, the H3K27ac decrease upon *FBXO22* depletion mirrors the epigenetic changes observed during STB differentiation. Genome-wide mapping via H3K27ac ChIP-seq and H3K9me2 CUT&Tag revealed an overall reduction in H3K27ac and H3K9me2 signals upon *FBXO22* depletion (Fig. 5B and C). However, key STB-upregulated genes, including *CTSD, ERVFRD-1, GCM1, CGB7*, and *SDC1*, paradoxically showed markedly increased H3K27ac enrichment at their promoters (Fig. 5D and F, and Supplementary Dataset S4), whereas STB-downregulated genes exhibited a marked loss of H3K27ac (Fig. 5E). Motif analysis of H3K27ac peaks identified potential transcription factor binding sites in control hTSCs, including *TEAD1, TEAD3, TEAD4, GATA2, GATA3*, and *OVOL2*, which are critical for maintaining trophoblast proliferation (Supplementary Fig. S9B).

H3K9me2 peaks were distributed in the promoter regions, gene body regions, and distal intergenic regions (Supplementary Fig. S9C). By integrating H3K27ac ChIP-seq, H3K9me2 CUT&Tag, and RNA-seq data, we identified 142 potential genes co-regulated by FBXO22 through both H3K27ac and H3K9me2 modifications (Supplementary Fig. S9D). GO enrichment analysis of these 142 genes revealed significant associations with biological processes such as regulation of cell migration, cell differentiation, epithelial development, and angiogenesis (Supplementary Fig. S9E). ChIP-qPCR validation confirmed that *FBXO22* knockdown increased H3K27ac at STB marker genes (*CTSD, ERVFRD-1, GCM1, CGB7*, and *SDC1*), while decreasing these modifications at stemness-related genes (*GATA3 and TEAD4*) (Fig. 5G and Supplementary Fig. S9F). Moreover, depletion of *FBXO22* led to a pronounced decrease in the repressive H3K9me2 modifications on STB marker genes (Fig. 5F and H), whereas no statistically significant differences were detected at stemness-related genes (Supplementary Fig. S9G). Luciferase reporter assays further demonstrated that *FBXO22* knockdown enhanced the promoter activity of STB-related genes (Fig. 5I) but suppressed that of stemness-related genes (Supplementary Fig. S9H). Collectively, these findings demonstrate that *FBXO22* deficiency remodels the chromatin landscape, creating a chromatin environment that selectively activates STB-specific gene programs while repressing stemness.

### FBXO22 overexpression attenuates trophoblast syncytialization *in vivo*

**Figure 6.**
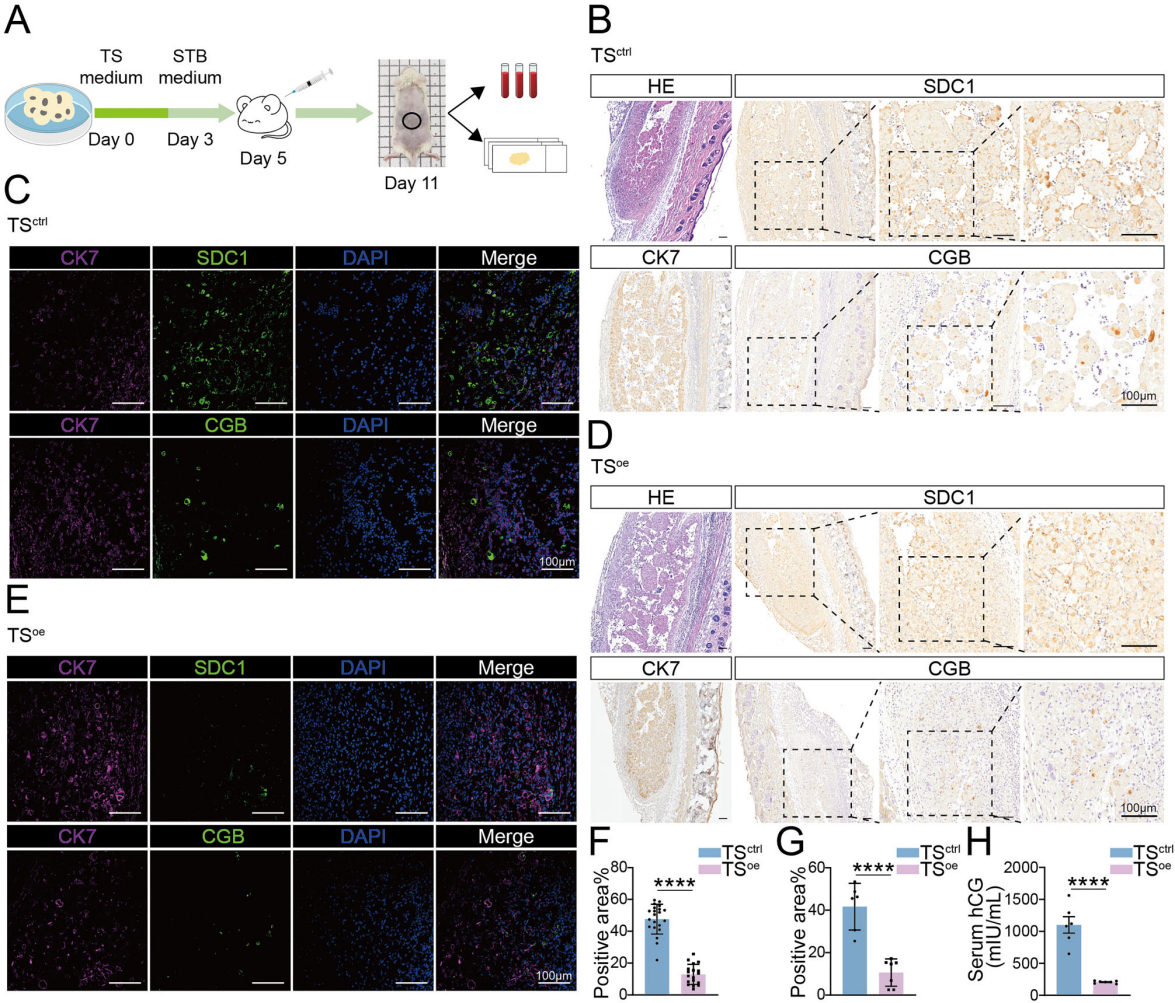
FBXO22 overexpression attenuates *in vivo* trophoblast syncytialization. (**A**) Schematic diagram of the protocol for engrafting hTSCs into NOD-SCID mice. CK7-positive trophoblast cells were used to define the graft area. SDC1 positive and CGB positive trophoblast cells were used to define the STBs area. (**B**) Immunohistochemical staining of a TS^ctrl^-derived graft showing CK7 (trophoblast marker), SDC1 and CGB (STB markers) staining. (**C**) Immunofluorescence images of a TS^ctrl^-derived graft. (**D**) Immunohistochemical staining of a TS^oe^-derived graft. (**E**) Immunofluorescence images of a TS^oe^-derived graft. (**F**) Analysis of STBs/graft area% by immunohistochemical staining across the tissue slices; n = 3 for every graft. (**G**) Analysis of STBs/graft area% by immunohistochemical staining across the tissue slices; n = 6 for TS^ctrl^, and n = 7 for TS^oe^. (**H**) Serum hCG levels in mice xenografted with TS^ctrl^ and TS^oe^; n = 6 for TS^ctrl^, and n = 7 for TS^oe^. Data are means ± SEM; ****P < 0.0001 by unpaired two-tailed *t* test. The blood tube images and the syringe image in Fig. 6A were adapted from SciDraw under the Creative Commons license CC BY 4.0.

To validate our findings in a physiological context, we utilized an *in vivo* xenograft model, a well-established system for studying human trophoblast differentiation [10, 13]. hTSCs transduced with control vector (TS^ctrl^) or a vector overexpressing *FBXO22* (TS^oe^) were subcutaneously injected into immunodeficient NOD-SCID mice (Fig. 6A). After 7 days, xenografts were harvested and analyzed for evidence of trophoblast differentiation. The serum hCG levels were measured, and xenografted tissues were subjected to immunofluorescence and immunohistochemistry analysis to assess the extent of trophoblast syncytialization (Fig. 6A). In TS^ctrl^ xenografts, we observed widespread and robust formation of multinucleated STBs, consistent with normal syncytialization patterns (Fig. 6B and C). In contrast, the formation of multinucleated STBs was markedly diminished in TS^oe^ xenografts (Fig. 6D and E). Quantitative analysis further confirmed a significantly lower ratio of STB area to total graft area in the TS^oe^ group (Fig. 6F and G), indicating a suppression of STB differentiation and expansion when FBXO22 levels are elevated. Consistent with impaired syncytialization, mice bearing TS^oe^ grafts exhibited significantly lower serum levels of hCG, this decrease highlighting a clear disruption of STB functional maturation (Fig. 6H). Together, these *in vivo* data provide strong physiological support for our cell culture findings and demonstrate that elevated FBXO22 expression is sufficient to compromise trophoblast syncytialization *in vivo*. This underscores FBXO22 as a pivotal negative regulator of trophoblast syncytialization, reinforcing its potential relevance to placenta-associated pregnancy disorders.

### FBXO22 downregulation and subsequent CoREST accumulation are linked to RPL

**Figure 7.**
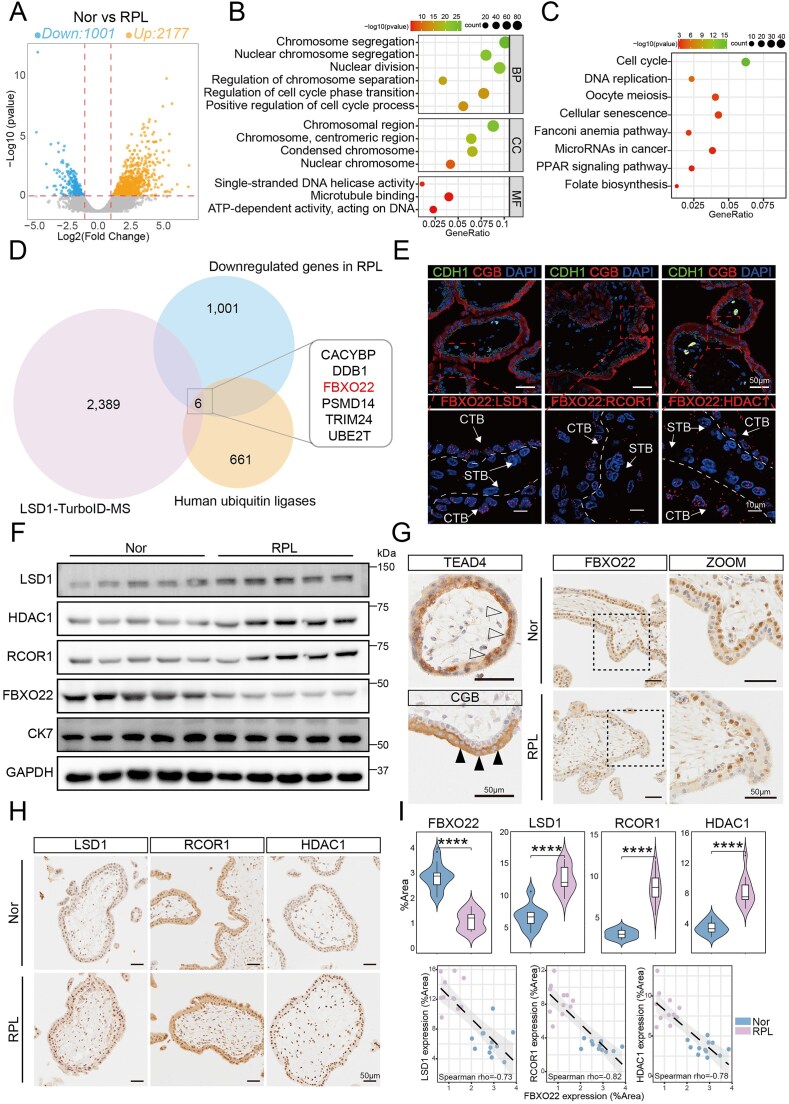
FBXO22 deficiency leads to CoREST complex accumulation and contributes to RPL. (**A**) Volcano plot of genes differentially expressed in first-trimester villi from normal pregnancies and in RPL patients (*P* < 0.05, log_2_ (fold change) > 0.8). (**B**) GO enrichment analysis of genes down-regulated in RPL patients. (**C**) Kyoto Encyclopedia of Genes and Genomes (KEGG) enrichment analysis of genes downregulated in RPL patients. (**D**) Venn diagram showing the overlap between the LSD1-interactome, transcriptome downregulated genes, and human ubiquitin ligases. (**E**) PLA assay showing the proximity between FBXO22 and CoREST in the early villous tissues. CDH1 and CGB were used as markers for the CTBs and STBs, respectively. The data presented are representative of at least three independent experiments. (**F**) Western blot analysis of FBXO22 and CoREST complex protein levels in normal pregnancies and in RPL patients. Cytokeratin 7 (CK7) was used as the trophoblast marker control; *n* = 5. (**G**) Immunohistochemical analysis of FBXO22 in the early villous tissues from normal pregnancies and from RPL patients. TEAD4 and CGB were used as markers for CTBs and STBs, respectively. (**H**) Immunohistochemical analysis of the CoREST complex in the early villous tissues from normal pregnancies and from RPL patients. (**I**) The expression levels and correlation analysis of FBXO22 and the CoREST complex in early villous tissues from normal pregnancies and from RPL patients; *n* = 12 for Nor, and *n* = 10 for RPL. Data are means ± SEM; ****P < 0.0001 by unpaired two-tailed *t* test.

Our data indicate that FBXO22 plays a critical role in early placental development, a period vulnerable to pregnancy failure. We therefore performed RNA-seq on early placental villi from normal pregnancies and RPL patients. Compared with the normal group, RPL villi exhibited widespread transcriptional dysregulation, with 2177 genes upregulated and 1001 genes downregulated (Fig. 7A and Supplementary Dataset S2). GO enrichment of the downregulated genes revealed significant enrichment in processes that included chromosome segregation, cell cycle regulation, and DNA replication, indicating the impaired proliferative capacity of trophoblast cells in RPL patients (Fig. 7B). Kyoto Encyclopedia of Genes and Genomes (KEGG) pathway analysis revealed that the downregulated genes were enriched in pathways involved in cell cycle and DNA replication (Fig. 7C). Notably, this transcriptomic signature closely aligns with the known functions of the CoREST transcriptional repressor complex. Comparing our LSD1 interactome with the RPL transcriptome, and the human ubiquitin ligases, we identified FBXO22 as a key candidate E3 ligase the loss of which could explain the observed gene expression changes (Fig. 7D). In villous tissues, the interaction between FBXO22 and CoREST was confirmed in the nuclei of CTBs via PLA (Fig. 7E, and Supplementary Fig. S10A and B). Strikingly, villous tissues from RPL patients showed markedly reduced *FBXO22* expression at both the mRNA and protein levels. In contrast, the proteins of the CoREST complex were significantly elevated despite no changes in mRNA levels, pointing to impaired protein degradation (Fig. 7F, and Supplementary Fig. S10C and D). In addition, the protein levels of GCM1 and CGB were increased in RPL villous samples with reduced FBXO22 expression (Supplementary Fig. S10E). Immunohistochemical analysis confirmed significantly elevated CoREST protein levels in RPL villi, and revealed a clear inverse correlation with FBXO22 expression (Fig. 7G–I). Collectively, these results indicate that reduced FBXO22 expression and consequent pathological accumulation of the CoREST complex are key molecular features associated with RPL.

## Discussion

Successful placental development depends on the precise regulation of trophoblast lineage specification and differentiation, processes essential for maternal–fetal exchange and early pregnancy maintenance. Disruption of trophoblast function has been implicated in multiple pregnancy complications, including RPL. In this study, we demonstrate that the nuclear-localized E3 ubiquitin ligase FBXO22 maintains the balance between trophoblast stem cell self-renewal and syncytialization. Loss of *FBXO22* in hTSCs led to aberrant and premature STB formation, impairing early placental development. Mechanistic analyses revealed that FBXO22 directly interacted with the CoREST transcriptional repressor complex, modulating histone modifications, including H3K27ac and H3K9me2. *FBXO22* deficiency resulted in increased nuclear LSD1 and HDAC1 levels, which in turn altered H3K27ac and H3K9me2 enrichment on the promoters of STB-associated genes, promoting their expression. Importantly, we observed a marked reduction of FBXO22 expression specifically in the nuclei of CTBs from RPL patients. Collectively, these findings have uncovered a previously unrecognized nuclear ubiquitination–epigenetic regulation axis that safeguards early trophoblast development and highlights the essential role of FBXO22 in the maintenance of early pregnancy.

The establishment of hTSCs and their differentiation into STBs depend on a complex regulatory network involving epigenetic modifications and multiple signaling pathways. Previous studies have shown that activation of Wnt and EGF signaling, combined with inhibition of TGF-β, HDAC, and ROCK, is critical for maintaining hTSC multipotency [10]. Transcription factor MSX2 helps preserve stemness and prevents premature STB differentiation by recruiting the SWI/SNF canonical BAF subcomplex to suppress H3K27ac modifications [46]. In contrast, GCM1 functions as a master driver of syncytialization by activating *ERVW-1* (Syncytin-1) and *ERVFRD-1* (Syncytin-2), while antagonizing the stemness factor ΔNp63α [47, 48]. Several signaling pathways also fine-tune STB development: the cAMP–GCM1-–Syncytin positive feedback loop amplifies fusion protein expression and accelerates syncytialization [49], Wnt/β-catenin signaling enhances *GCM1* transcription [50], whereas hypoxia inhibits GCM1 expression, and blocks STB formation [51]. In addition, TFEB has been shown to directly promote *ERVFRD-1* expression, suggesting that auxiliary regulatory mechanisms beyond the GCM1-driven network cooperate to ensure proper syncytialization [33]. Notably, a study using human haploid induced trophoblast stem cells derived from human haploid embryonic stem cells identified EBI3 as a critical regulator of STB specification [52]. Collectively, these studies provide a preliminary framework for understanding the transcriptional and signaling regulation of STB differentiation. However, the contribution of protein homeostasis, particularly ubiquitination-mediated regulation, remains poorly defined. Our study addresses this knowledge gap by demonstrating that FBXO22, a nuclear-localized E3 ubiquitin ligase downregulated in CTBs from RPL patients, is critical for preserving hTSC stemness and preventing uncontrolled STB differentiation. This positions FBXO22-dependent nuclear ubiquitination as an integral and previously overlooked component of trophoblast lineage regulation.

FBXO22 is the substrate recognition subunit of the SCF ubiquitin ligase complex and has a broad substrate range, including cell cycle regulators, tumor suppressors, immune regulatory molecules, membrane proteins, and key signaling nodes [24, 26, 53–56]. Depending on the tissue and context, FBXO22 can exhibit both oncogenic and tumor-suppressive roles, suggesting that it functions as an environment- and tissue-specific regulator of protein homeostasis rather than a unidirectional factor. Although FBXO22 has been reported to localize to both the nucleus and cytoplasm, its nuclear function remains poorly understood. Furthermore, prior studies have largely focused on tumor models, leaving its role in pregnancy largely unexplored. In the present study, we found *FBXO22* deficiency in hTSCs drives premature STB formation, whereas overexpression of *FBXO22* under STB culture conditions suppresses normal syncytialization. These findings indicate that dysregulated *FBXO22* can directly disrupt the balance between hTSC proliferation and STB differentiation. Notably, FBXO22 has been reported to promote tumorigenesis by ubiquitinating and degrading nuclear PTEN [24]. Strikingly, our study extends this paradigm by identifying FBXO22 as a direct regulator of a nuclear epigenetic complex—the CoREST complex. FBXO22 selectively ubiquitinates the CoREST complex, modulating its stability thereby reshaping histone modifications, such as H3K27ac and H3K9me2. Apart from FBXO22, other candidate E3 ubiquitin ligases identified by LSD1-TurboID may also participate in the regulation of placental development. STUB1 has been reported to regulate TFEB protein stability through the ubiquitin-proteasome pathway [57], whereas TFEB contributes to STB formation by regulating the expression of Syncytin-1 and Syncytin-2 [33, 44, 58]. Other candidates also have been implicated in trophoblast or placental biology. Cbl regulates trophoblast invasion by mediating Met ubiquitination, MULE participates in Mcl-1 and p53 degradation and has been linked to placental stress responses, and Hectd1 is required for placental labyrinth development [59–61]. Collectively, FBXO22 is not limited to cytoplasmic protein homeostasis but also acts as a guardian of nuclear protein stability. This previously unrecognized nuclear function positions FBXO22 as a central node linking ubiquitination, epigenetic regulation, and cell fate determination, extending our understanding of the mechanisms controlling trophoblast differentiation and placental development.

The CoREST complex was initially identified as a co-repressor of REST, with RCOR1 serving as a scaffold for HDAC1/2 and LSD1 to remove active chromatin marks, including histone acetylation and H3K4 methylation [62, 63]. However, increasing evidence suggests that the chromatin regulatory effect of the CoREST complex is not fixed, but instead depends on its subunit composition, the local chromatin environment, and the transcription factor networks with which it cooperates [64–67]. Under *FBXO22*-deficient conditions, the phenotype caused by CoREST component accumulation is interpreted as a state in which global repression coexists with locus-selective release, rather than as comprehensive conversion from repression to activation. Specifically, *FBXO22* depletion led to abnormal HDAC1 accumulation accompanied by a reduction in global H3K27ac. Previous studies have shown that HDAC1/2 activity is required for syncytialization, and that histone acetylation marks, including H3K27ac and H3K14ac, undergo a transient decline during the CTB-to-STB transition [19]. Together with our findings, this suggests that the HDAC1-induced decrease in H3K27ac is more likely to destabilize the chromatin environment required for stemness-related genes, including *TEAD4* and *GATA3*, thereby promoting exit from the hTSC state and creating a permissive window for fate transition. Moreover, the increase in LSD1 induced by *FBXO22* deficiency predominantly affected H3K9me2 rather than its canonical substrate H3K4me2. More importantly, this reduction in H3K9me2 occurred preferentially at STB-related loci, including *CTSD, SDC1, ERVFRD-1, GCM1*, and *CGB7*. Previous work has shown that, during syncytialization, LSD1 removes repressive H3K9me2 at loci such as *ERVW-1, CGA*, and *CGB*, and cooperates with GATA2 to promote RNA polymerase II recruitment, thereby activating the STB program [17]. Thus, under *FBXO22*-deficient conditions, HDAC1-mediated global hypoacetylation may weaken the stemness program, whereas LSD1-mediated loss of H3K9me2 relieves local repression at STB-related loci.

It is also important to emphasize that HDAC1 and LSD1 do not function independently within the CoREST complex. Structural and biochemical studies have shown that their catalytic activities are interdependent, such that perturbation of one component can compromise the function of the other [68–71]. Our pharmacological results are consistent with this view, as inhibition of either HDAC1 or LSD1 alone was sufficient to suppress STB formation. These findings suggest that, during syncytialization, HDAC1 and LSD1 are not simply opposing regulators, but instead cooperate at distinct regulatory layers to shape the chromatin state required for fate transition. In addition, given that CoREST lacks intrinsic sequence-specific DNA-binding activity, its selective localization at STB loci is more likely to depend on trophoblast-specific transcription factors that already occupy these regions. In other studies, CoREST has been shown to be targeted to specific chromatin loci through interactions with DNA-binding transcription factors. For example, CoREST complex was originally identified as a co-repressor for REST, GFI1/GFI1B can recruit the LSD1–CoREST complex, and Snail1 can form a Snail1–LSD1–CoREST ternary complex through its SNAG domain and direct it to target promoters or other regulatory chromatin regions [63, 67, 72]. In the studies for STB differentiation, several key transcription factors have already been identified that directly occupy and activate STB-related loci. Among them, GCM1 directly binds to and activates canonical STB-related genes, including *ERVW-1* and *ERVFRD-1* [73, 74]. In addition, TFEB undergoes nuclear translocation during STB formation and directly binds the promoters of *ERVW-1, ERVFRD-1*, and steroidogenic genes [44]. BHLHE40 can also form a complex with GATA2/GATA3, enhance their chromatin occupancy at loci such as *CGB* and *HSD17B1*, and maintain locally active chromatin states marked by H3K27ac and H3K4me3 [75]. Based on these findings, together with our motif analysis, we speculate that under *FBXO22*-deficient conditions, accumulated CoREST components may be selectively recruited or retained at STB-associated loci by trophoblast transcription factors such as TEAD1/3/4, GATA2/3, OVOL2, and TFAP2C, which merits further investigation.

Human reproduction is inherently inefficient, with approximately 15% of pregnancies ending in early miscarriage [76, 77], and about 5% of women experience RPL [1, 2]. Despite placental pathology being a key factor, its molecular mechanisms remain unclear. In this study, we found that FBXO22 levels were markedly reduced in CTBs from RPL patients, leading to pathological accumulation of the CoREST complex. This imbalance compromises CTB self-renewal and promotes premature STB differentiation, disrupting villous architecture and providing a mechanistic basis for early pregnancy loss. While placental disorders vary clinically, trophoblast fate imbalance appears to be a common mechanism: RPL features deficient CTB stemness and inappropriate syncytialization, whereas preeclampsia (PE) and intrauterine growth restriction (IUGR) involve inadequate STB formation and restricted EVT invasion [78, 79]. Interestingly, we observed that overexpression of FBXO22 inhibited STB formation, suggesting a potential role in PE, where defective syncytialization is a hallmark. Moreover, FBXO22-regulated H3K27ac peaks were enriched for potential TFAP2C-binding motifs, consistent with reports that TFAP2C is essential for EVT differentiation and that its dysregulation may lead to shallow implantation and defective spiral artery remodeling [80]. Together, these results point to a broader role for FBXO22 in placental insufficiency syndromes, although whether it directly regulates EVT invasion requires further investigation.

There are limitations that should be acknowledged in our study. First, our conclusions are primarily based on analyses of early human villous tissues and *in vitro* trophoblast stem cell models, which, although informative, cannot fully recapitulate the complexity of placental development *in vivo*. In addition, currently available animal models do not faithfully reproduce key features of human placentation, which further limits *in vivo* validation of the mechanism identified here. Second, the upstream cause of FBXO22 downregulation in RPL is likely multifactorial, and future studies will be needed to define the pathological signals that regulate FBXO22 expression at the maternal–fetal interface. A more comprehensive understanding of how FBXO22 is transcriptionally and post-transcriptionally controlled within the broader ubiquitin regulatory network will be important for defining how it is integrated into trophoblast fate regulation. Third, the molecular basis by which FBXO22 recognizes and regulates the CoREST complex remains incompletely understood. In particular, it remains unclear which subunits of the CoREST complex are directly recognized by FBXO22, whether substrate recognition depends on a specific degron or phosphorylation-dependent interaction, and which transcription factors mediate the locus-specific recruitment of the CoREST complex. Fourth, our work primarily centers on the molecular basis of STB differentiation, leaving the potential roles of FBXO22 in EVT invasion and placental vascular remodeling to be further explored. Finally, while the clinical samples analyzed are representative, the cohort size is relatively limited, and future cross-disease cohort studies coupled with mechanistic validation will be essential to delineate both the shared and distinct roles of FBXO22 in placental pathologies such as RPL, PE, and IUGR.

In summary, our findings reveal a pivotal role for FBXO22 in human trophoblast development. By mediating the ubiquitination of the CoREST complex, FBXO22 preserves the stability of histone modifications, thereby maintaining the delicate balance between trophoblast stem cell self-renewal and syncytialization. These findings provide new mechanistic insights into placental development and the pathogenesis of RPL, emphasizing the critical interplay between protein homeostasis and epigenetic regulation in early pregnancy. Importantly, our work raises the possibility that targeting FBXO22-regulated pathways could offer new strategies for diagnosing or treating placental insufficiency and RPL.

## Supplementary Material

gkag557_Supplemental_Files

## Data Availability

The raw sequence data reported in this paper have been deposited in the Genome Sequence Archive at the National Genomics Data Center under project HRA014079 and HRA014155. The data, analytical methods, and research materials in this article will be made available to other researchers upon reasonable request from the corresponding authors.
