# Supplementary material for: Reduced FBXO22 skews human trophoblast fate equilibrium toward syncytialization via polyubiquitinating the CoREST complex
Source: Nucleic Acids Res. 2026 Jun 4;54(10):gkag557. doi: 10.1093/nar/gkag557 (PMC13234505; doi:10.1093/nar/gkag557)
Supplement: gkag557_Supplemental_Files [file gkag557_supplemental_files.zip › 290526035038_Supplementary_materials.pdf]

Supplementary information

Reduced FBXO22 skews human trophoblast fate equilibrium toward syncytialization via polyubiquitinating the CoREST complex

Hongli Li[1,□], Guangmin Song[1,□], Linwei Zhou[1], Man Zhang[1], Yun Li[1], Xinmi Liu[1], Xing Wang[1], Li Yang[1], Xinyi Tao[1], Richard D. Cannon[2], Richard Saffery[3], Boris Novakovic[3], Hongbo Qi[1], Xiaobo Zhou[1,*], Hua Zhang[1]

[1]Department of Obstetrics and Gynecology, Chongqing Key Laboratory of Maternal and Fetal Medicine/Joint International Research Laboratory of Reproduction & Development, Ministry of Education/The Innovation and Talent Recruitment Base of Maternal-Fetal Medicine, The First Affiliated Hospital of Chongqing Medical University, No.1 Youyi Rd, Yuzhong District, Chongqing, 400016, China.

[2]Department of Oral Sciences, Sir John Walsh Research Institute, Faculty of Dentistry, University of Otago, Dunedin 9016, New Zealand

[3]Molecular Immunity, Infection and Immunity Theme, Murdoch Children's Research Institute, Royal Children's Hospital, Parkville, VIC 3052, Australia

[□]These authors contributed equally to this paper.

[*]Correspondence: xiaobo_zhou@cqmu.edu.cn

This file includes:

Supplementary Figures 1-10

Supplementary Tables 1-3

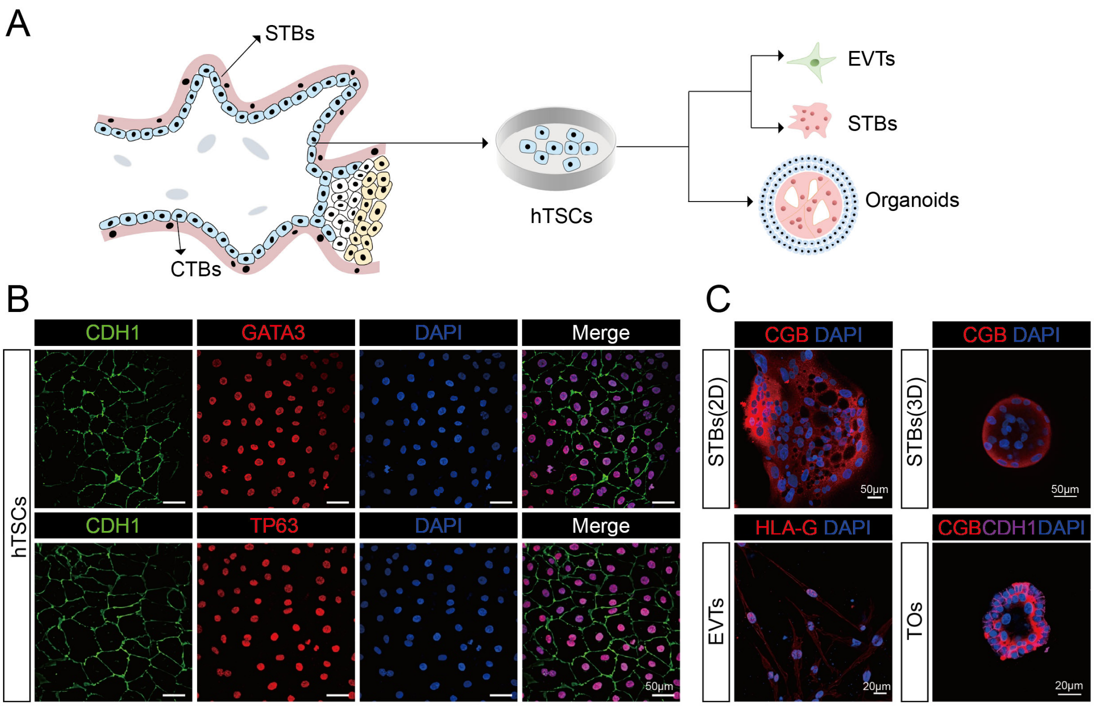

Figure S1. Establishment of the hTSC culture and STB differentiation models.

(**A**) Schematic diagram of hTSC derivation and differentiation.

(**B**) Immunofluorescent staining of hTSCs for canonical stem cell markers CDH1, GATA3, and TP63.

(**C**) Immunofluorescence staining for trophoblast differentiation: CGB for STBs (2D and 3D), HLA-G for EVTs, and CGB with CDH1 for TOs.

The images presented are representative of at least three independent experiments. CTB: cytotrophoblast; STBs, syncytiotrophoblasts; hTSCs, human trophoblast stem cells; EVTs, extravillous trophoblasts; TOs, trophoblast organoids.

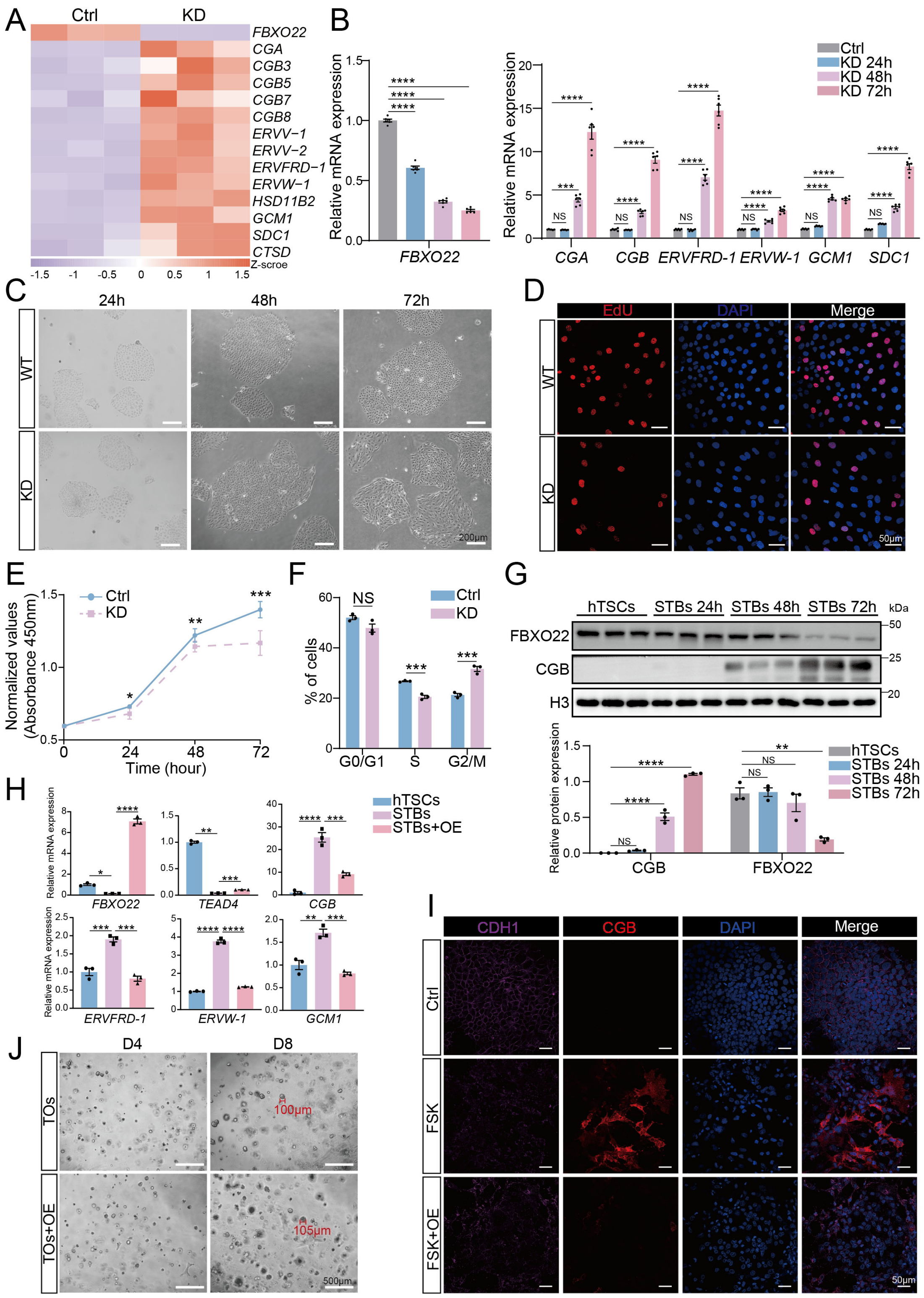

Figure S2. FBXO22 is a critical regulator of trophoblast cell fate.

(**A**) Heatmap of Z-score for STB markers in control and *FBXO22*-Knockdown (*FBXO22*-KD)hTSCs.

(**B**) RT-PCR analysis of STB markers expression in control and *FBXO22*-KD hTSCs at 24, 48

and 72 h. The values were normalized to *H2A*; n=6.

(**C**) Representative bright field images showing the cell morphology of control and *FBXO22*-KD hTSCs.

(**D**) Representative EdU immunofluorescence images of control and *FBXO22*-knockdown hTSCs.

(**E**) The CCK8 assay of cell proliferation in control and *FBXO22*-KD hTSCs at 0, 24, 48 and 72 h. n=3.

(**F**) Cell cycle assay of the distribution of cells in G0/G1, S, and G2/M phases in control and *FBXO22*-KD hTSCs. n=3. NS, no significance.

(**G**) Western blot analysis of FBXO22 and CGB protein levels in hTSCs and hTSCs-derived STBs for 24, 48 and 72 h. H3 was used as loading control; n=3.

(**H**) RT-PCR analysis of *FBXO22*, *TEAD4* and STB markers mRNA levels in STBs, STBs with *FBXO22* overexpression (STB+OE-*FBXO22*), and control hTSCs. The values are normalized to *H2A*; n=3.

(**I**) Immunofluorescence staining of CDH1 and hCG in BeWo cells treated for 48 h with DMSO, FSK (25 μM), or FSK combined with *FBXO22* overexpression. The data presented are representative of at least three independent experiments.

(**J**) Representative bright field images showing the morphology of *FBXO22*-overexpressing and control hTSC-derived organoids at day 4 and day 8.

Data are means ± SEM; *$p < 0.05$, **$p < 0.01$, ***$p < 0.001$, ****$p < 0.0001$ by unpaired two-tailed t test (E-F) and one-way ANOVA followed by Tukey's multiple comparisons test (B and G-H).

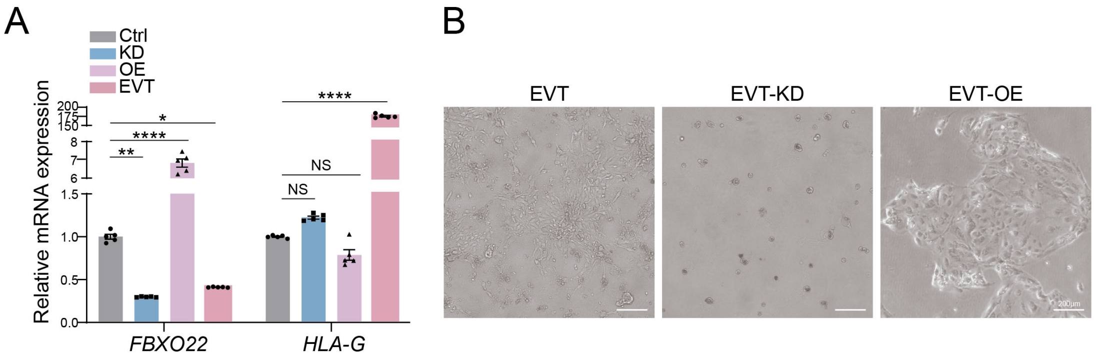

Figure S3. FBXO22 dysregulation in hTSCs is insufficient to induce EVT differentiation.

(**A**) RT-PCR analysis of *FBXO22* and *HLA-G* mRNA levels in control hTSCs, *FBXO22*-KD cells, *FBXO22*-OE cells and hTSCs-derived EVTs. The values were normalized to *H2A*; n=5. NS, no significance.

(**B**) Representative bright field images showing the morphology of hTSCs-derived EVTs (EVT), *FBXO22*-KD hTSCs-derived EVTs (EVT-KD) and *FBXO22*-OE hTSCs-derived EVTs (EVT-OE).

Data are means ± SEM; The images presented are representative of at least three independent experiments; *$p < 0.05$, **$p < 0.01$, ***$p < 0.001$, ****$p < 0.0001$ by one-way ANOVA followed by Tukey's multiple comparisons test.

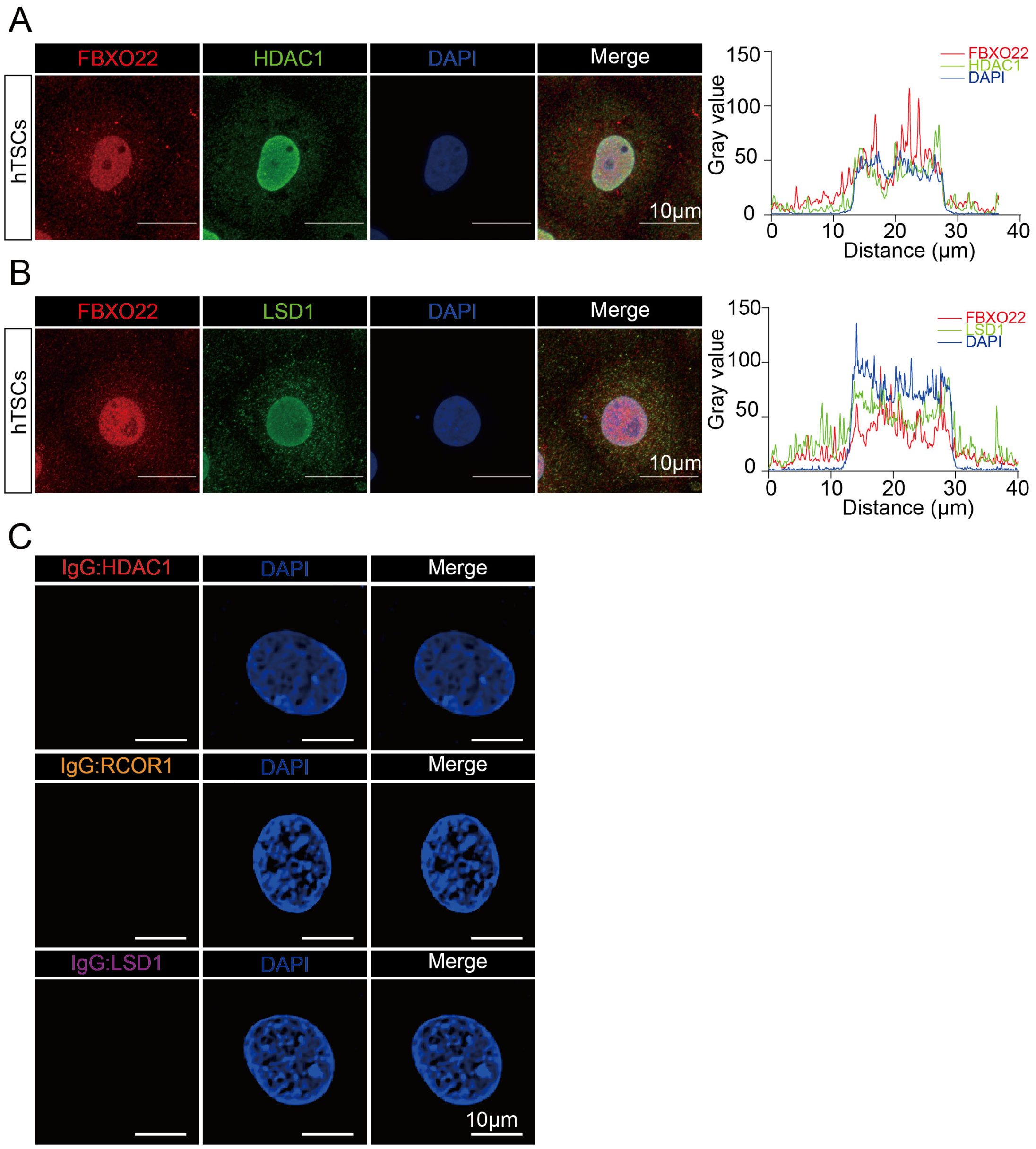

Figure S4. Nuclear co-localization of FBXO22 with the CoREST complex in hTSCs.

(**A**) Immunofluorescence staining showing the co-localization of FBXO22 and HDAC1 in hTSCs.

(**B**) Immunofluorescence staining showing the co-localization of FBXO22 and LSD1 in hTSCs.

(**C**) Negative (IgG) control for PLA assay in hTSCs.

The data presented are representative of at least three independent experiments.

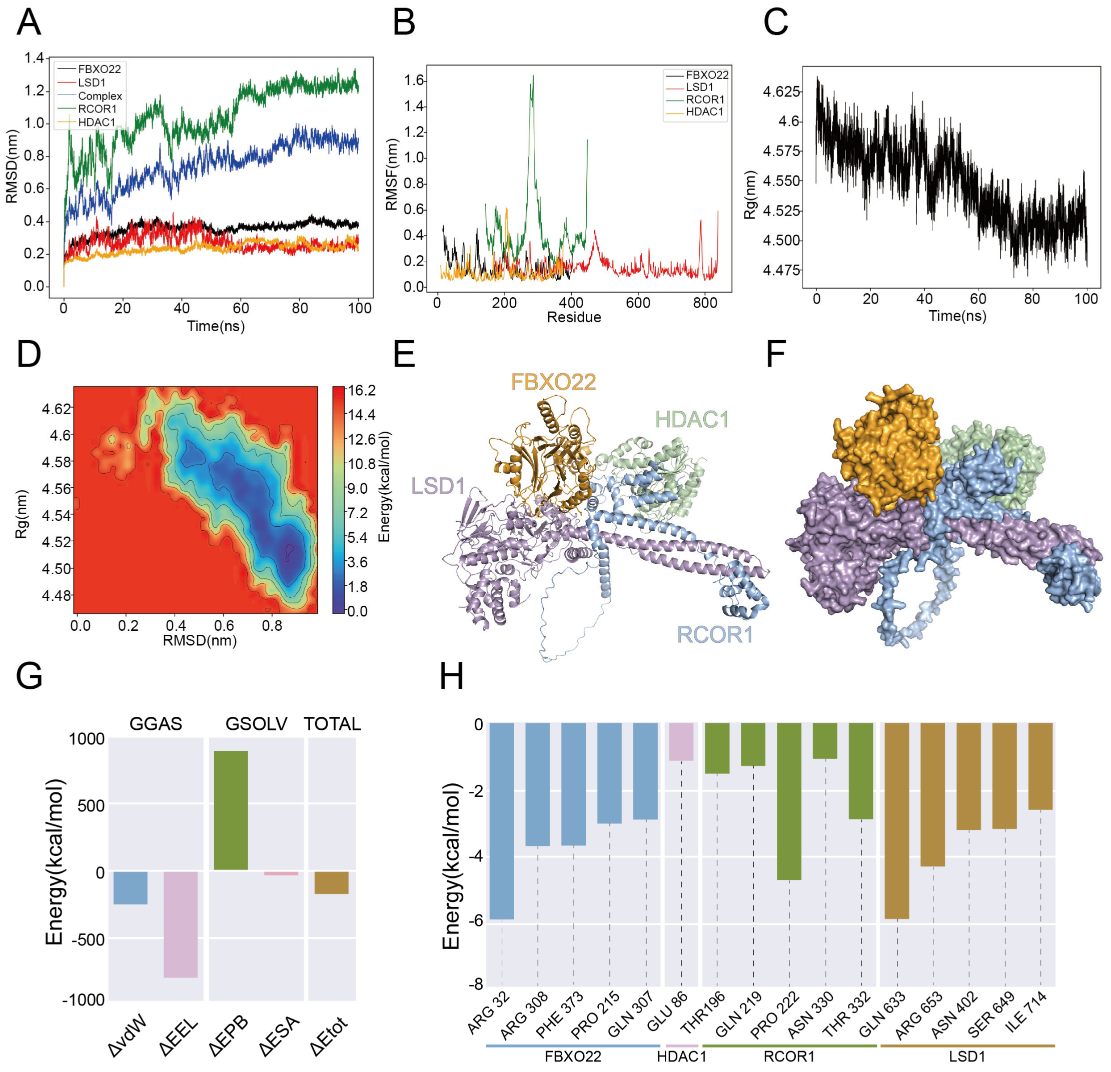

Figure S5. Molecular docking analysis of the FBXO22-CoREST interaction.

(**A-D**) The root-mean-square deviation (RMSD) (**a**), root-mean-square fluctuation (RMSF) (**b**), radius of gyration (Rg) (**c**) and free energy landscape (**d**) analysis of the FBXO22-CoREST binding complex.

(**E**) Binding surface image of the FBXO22-CoREST binding complex.

(**F**) 3D interaction interface diagram of the FBXO22-CoREST binding complex.

(**G**) Energetic component analysis of the FBXO22-CoREST binding complex.

(**H**) Bar chart of the FBXO22-CoREST binding complex binding free energy decomposition into residues.

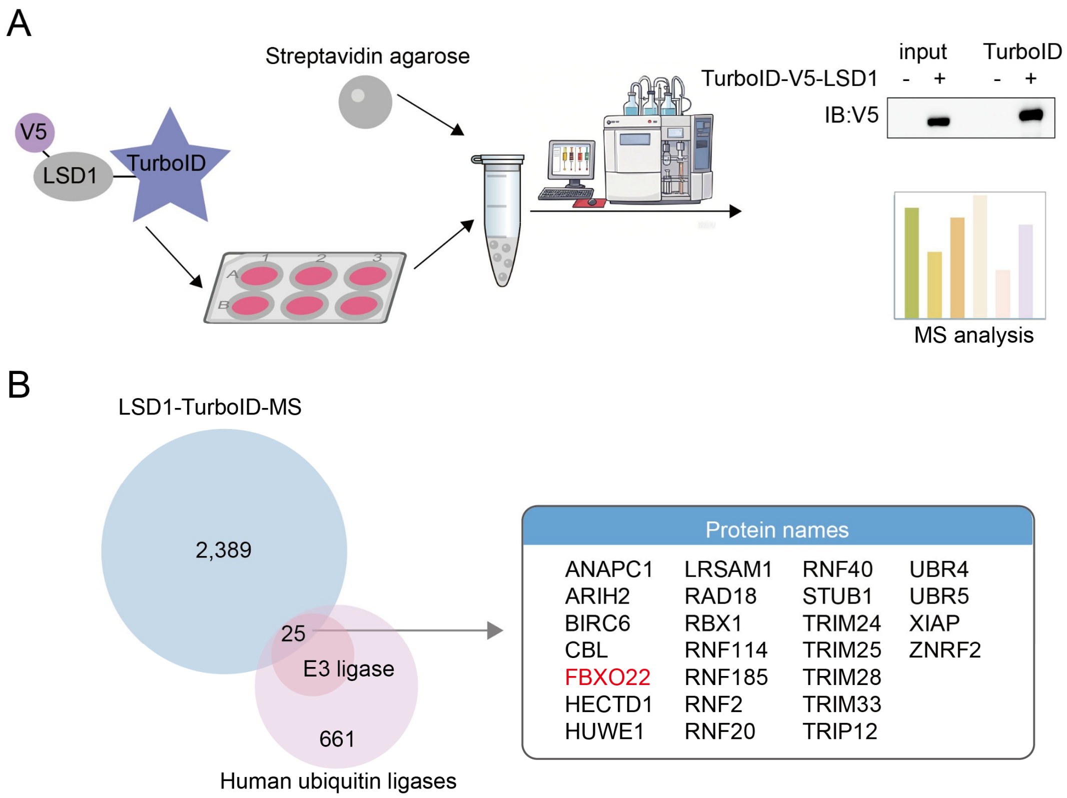

Figure S6. Identification of LSD1-interacting proteins by TurboID labeling.

(**A**) Schematic diagram showing the process for identifying LSD1-interacting proteins using TurboID labeling.

(**B**) Venn diagram of E3 ubiquitin ligases among LSD1-interacting proteins.

The Eppendorf tube image in Fig. S6A was adapted from SciDraw under the Creative Commons license CC BY 4.0.

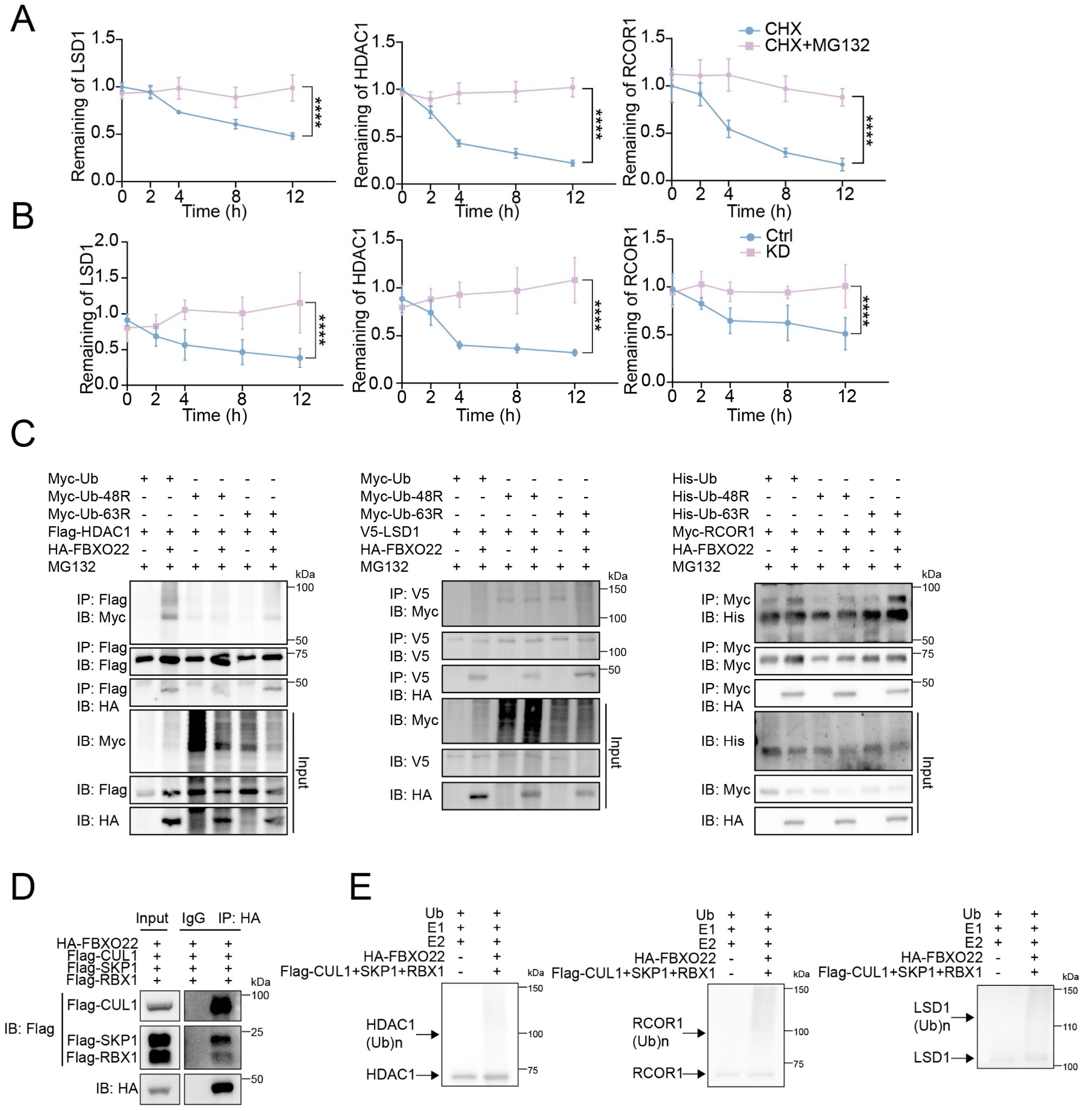

Figure S7. FBXO22-mediated CoREST ubiquitination.

(**A**) Quantitative analysis of CoREST complex protein levels in hTSCs treated with cycloheximide (CHX) or CHX combined with MG132; n=3.

(**B**) Quantitative analysis of CoREST complex protein levels in *FBXO22*-KD cells and control hTSCs following CHX treatment; n=3.

(**C**) Western blot analysis of CoREST complex ubiquitination in HEK293T cells co-transfected with WT-Ub, K48R or K63R plasmid.

(**D**) The expression of each subunit and the reconstitution of the SCF^{FBXO22} complex by IP from 293T cells are confirmed by western blotting

(**E**) The HDAC1, RCOR1, and LSD1 were immunoprecipitated from the reaction mixture with the indicated antibodies after the *in vitro* ubiquitination reaction, and ubiquitinated HDAC1,

RCOR1, and LSD1 were detected by western blotting using the UBCJ2 antibody (for mono- and poly-ubiquitinated conjugates).

Data are means ± SEM; The data presented are representative of at least three independent experiments; *$p < 0.05$, **$p < 0.01$, ***$p < 0.001$, ****$p < 0.0001$ by unpaired two-tailed t test.

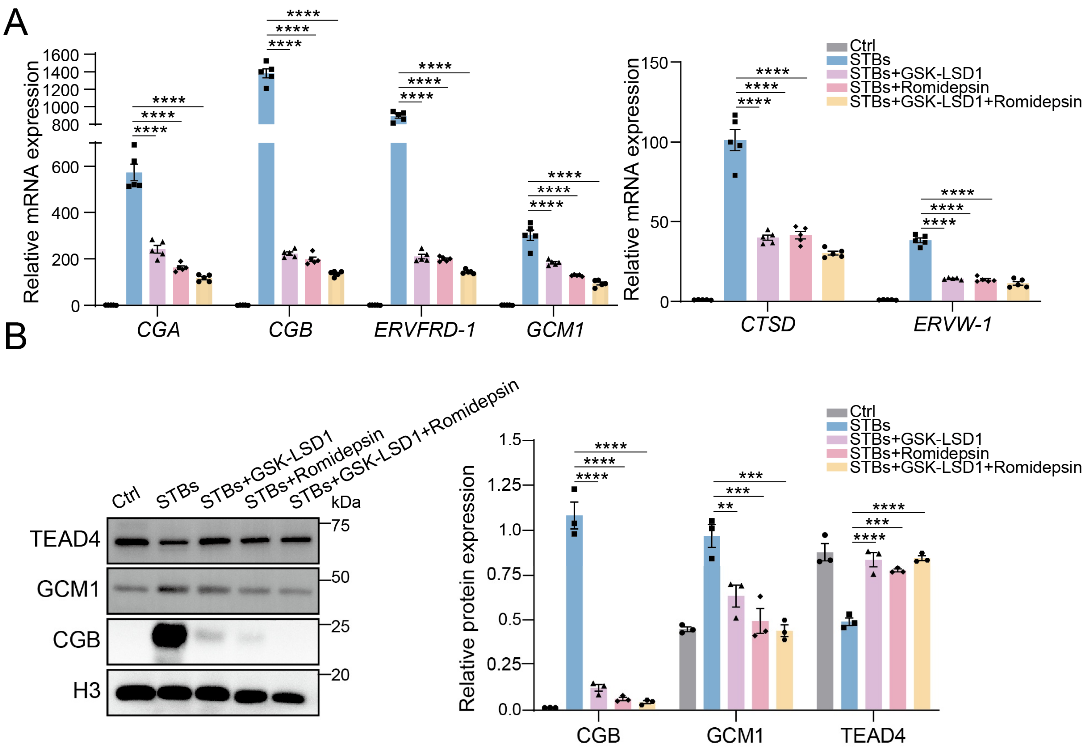

Figure S8. Inhibition of HDAC1 and LSD1 impairs proper syncytialization.

(**A**) RT-PCR analysis of STB marker genes mRNA expression in hTSCs, hTSCs-derived STBs, and hTSCs-derived STBs treated with Romidepsin and GSK-LSD1. The values were normalized to *H2A*; n=5.

(**B**) Western blot analysis of TEAD4 and GCM1 protein levels in hTSCs, hTSCs-derived STBs, and hTSCs-derived STBs treated with Romidepsin and GSK-LSD1. H3 was used as the loading control. The data presented are representative of at least three independent experiments.

Data are means ± SEM; *$p < 0.05$, **$p < 0.01$, ***$p < 0.001$, ****$p < 0.0001$ by one-way ANOVA followed by Tukey's multiple comparisons test.

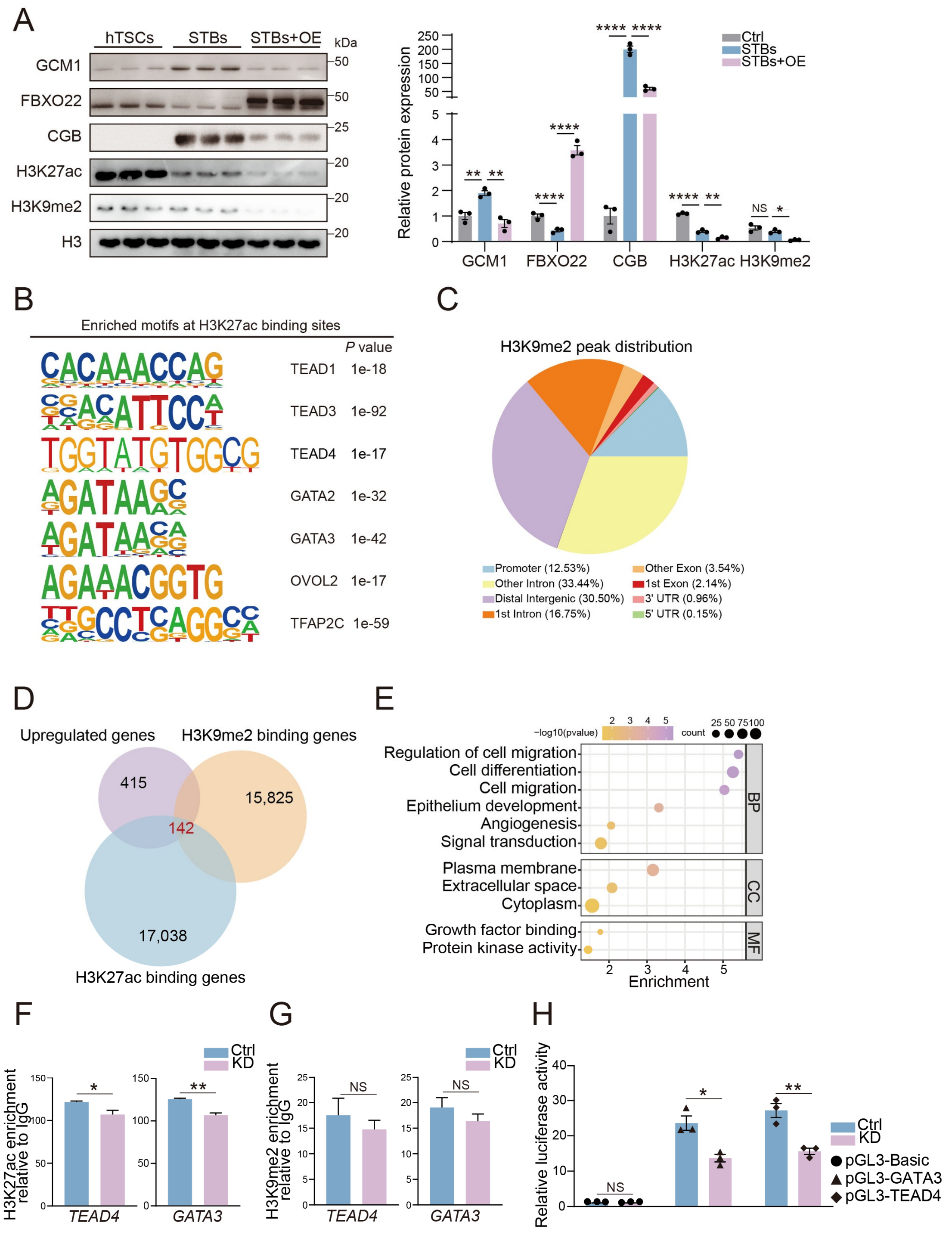

Figure S9. FBXO22 deficiency leads to dysregulation of H3K27ac and H3K9me2.

(**A**) Western blot analysis and quantification of FBXO22, GCM1, CGB, H3K27ac and H3K9me2 protein levels in STBs, STBs+OE-*FBXO22* and control hTSCs. H3 was used as the loading control; n=3.

(**B**) *De novo* motifs identified by HOMER at H3K27ac binding sites.

(**C**) Genomic distribution of H3K9me2 CUT&Tag peaks in hTSCs.

(**D**) Venn diagram showing the overlap among H3K9me2 binding peaks, H3K27ac binding peaks, and upregulated genes in *FBXO22*-KD hTSCs RNA-seq.

(**E**) GO enrichment analysis of FBXO22, H3K9me2 and H3K27ac target genes in hTSCs.

(**F**) ChIP-qPCR of H3K27ac modification in the promoter regions of stemness genes in *FBXO22*-KD cells and control hTSCs; n=3.

(**G**) ChIP-qPCR of H3K9me2 modification in the promoter regions of stemness genes in *FBXO22*-KD cells and control hTSCs; n=3. NS, no significance.

(**H**) Luciferase reporter assays of the activities of stemness gene promoters in *FBXO22*-KD and control cells; n=3. NS, no significance.

Data are means ± SEM; *$p < 0.05$, **$p < 0.01$, ***$p < 0.001$, ****$p < 0.0001$ by unpaired two-tailed t test (F-H) and one-way ANOVA followed by Tukey's multiple comparisons test (A).

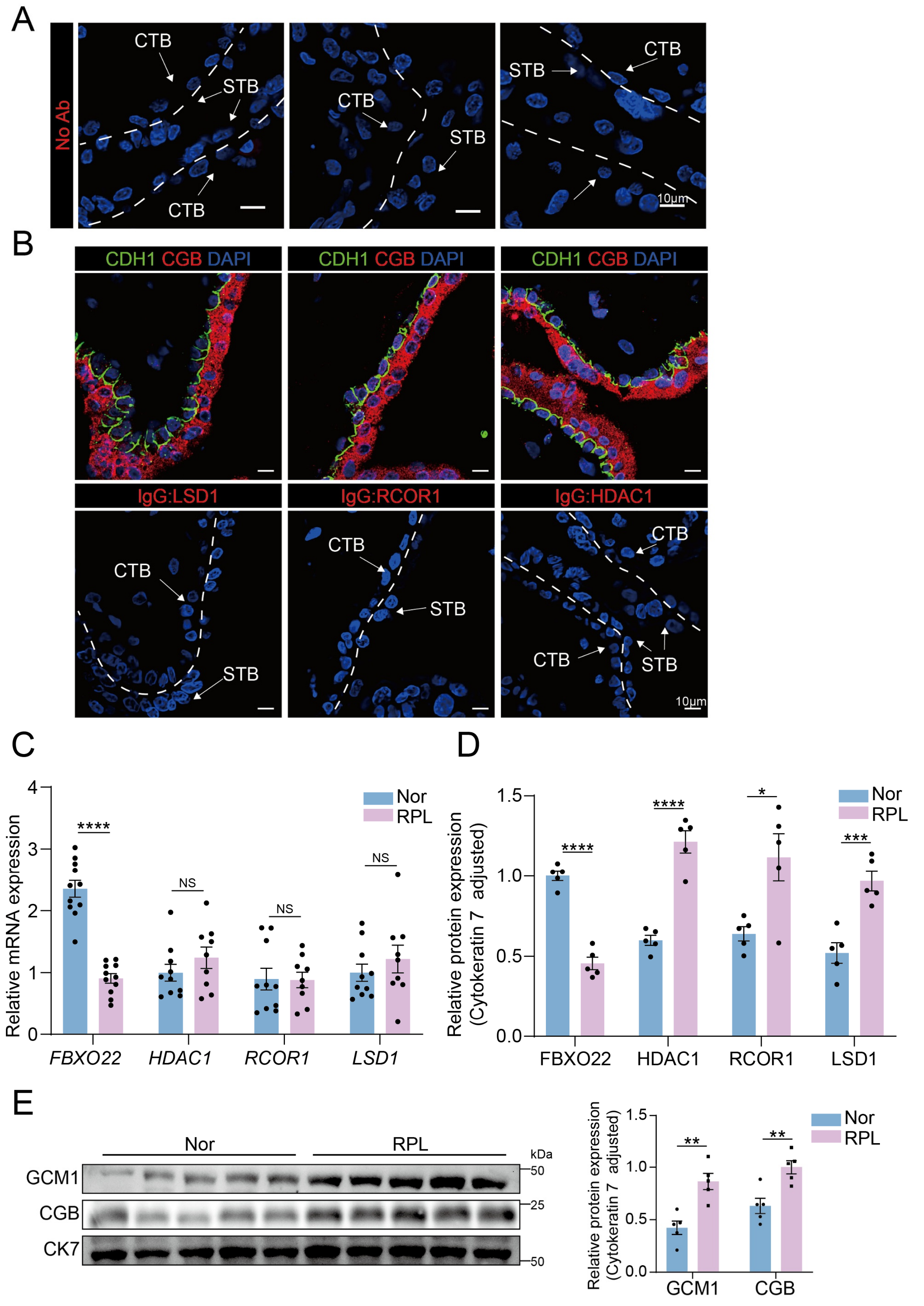

Figure S10. FBXO22 reduction leads to CoREST complex accumulation and is associated with RPL.

(**A**) Negative (no-primary) control for PLA assay in early villous tissue. No Ab: no primary antibody.

(**B**) Negative (IgG) control for PLA assay in early villous tissue. CDH1 and CGB were used as markers for the CTBs and STBs, respectively.

(**C**) RT-PCR analysis of *FBXO22*, *HDAC1*, *RCOR1* and *LSD1* mRNA levels in normal pregnancies and in RPL patients; n=10 for Nor, and n=9 for RPL. NS, no significance.

(**D**) Quantitative analysis of FBXO22 and CoREST complex protein levels in normal pregnancies and in RPL patients; n=5.

(**E**) Western blot analysis of GCM1 and CGB protein levels in normal pregnancies and in RPL patients. Cytokeratin 7 (CK7) was used as the trophoblast marker control; n=5.

Data are means ± SEM, the above experiments were repeated three times; *$p < 0.05$, **$p < 0.01$, ***$p < 0.001$, ****$p < 0.0001$ by unpaired two-tailed *t* test.

Table S1 The clinical characteristics of women enrolled in this study who provided early placental villous samples.

	Nor (n=12)	RPL(n=11)	*p*
Age (years)	33±1.628	29.82±1.242	0.1404[a]
Gestation age (weeks)	6.726±0.2178	7.065±0.1930	0.2609[a]
Previous unexplained pregnancy loss	0-1	2-5	<0.0001[b]
Parity	0-2	0-1	0.0079[b]
Chromosomal analysis	□ □	Normal	□ □

[a]Student t-test; [b]Mann-Whitney test.

Table S2 Chemicals and antibodies.

**Reagents**	**Company**	**Catalog Number**
Collagen IV	Corning	354233
DMEM/F12 medium	BasalMedia	L310KJ
High-glucose DMEM	ThermoFisher	11995073
Advanced DMEM/F12	ThermoFisher	12634010
Penicillin-Streptomycin (PS)	ThermoFisher	15140122
FBS for hTSCs	ThermoFisher	10099141
FBS for BeWo cells	ThermoFisher	A5256701
BSA	Sigma	A9418
2-mercaptoethanol	ThermoFisher	21985023
Insulin-Transferrin-Selenium-Ethanolamine (ITS-X)	BasalMedia	S452J7
L-ascorbic acid	Sigma	A5960
EGF	PeproTech	315-09-100
Y-27632	Selleck	S1049
CHIR99021	Selleck	S1263
A83-01	MedChemExpress	HY-10432A
SB431542	MedChemExpress	HY-10431
VPA	MedChemExpress	HY-10585
TrypLE Express	ThermoFisher	12604021
KnockOut Serum Replacement (KSR)	ThermoFisher	10828010
NRG1	CST	26941
Matrigel gel	Corning	356231
N2 supplement	ThermoFisher	17502048
B27 supplement	ThermoFisher	17504044

N-acetyl-L-cysteine	Merck	A9165
L-glutamine	ThermoFisher	25030081
R-spondin-1	MedChemExpress	HY-P7114
FGF-2	PeproTech	10018
HGF	PeproTech	10039
Prostaglandin E2	MedChemExpress	HY101952
Romidepsin	Selleck	S3020
GSK-LSD1	Selleck	S7574
Forskolin	MedChemExpress	HY-15371
MG132	MedChemExpress	S1748
3-Methyladenine (3-MA)	MedChemExpress	HY-19312
CHX	MedChemExpress	HY-12320
**Antibody**	**Company**	**Catalog Number**
FBXO22	Proteintech	13606-1-AP
LaminB1	Proteintech	12987-1-AP
β-catenin	Proteintech	51067-2-AP
β-actin	Proteintech	20536-1-AP
HDAC1	CST	34589
RCOR1	ABclonal	A3568
LSD1	ABclonal	A24121
TEAD4	GeneTex	GTX108750
CDH1	CST	3195
CK7	Abcam	AB181598
GCM1	Proteintech	21724-1-AP
CGB	ThermoFisher	MA514375
SDC1	Abcam	AB181789
H3	CST	9715

H3K27ac	CST	8173
H3K9me2	Active Motif	39041
H3K4me2	Abcam	Ab32356
GAPDH	Proteintech	60004-1-Ig
HA	Proteintech	51064-2-AP
Flag	Proteintech	66008-4-Ig
Myc	ABclonal	AE070
V5	Proteintech	14440-1-AP
His	CST	2365
Ub	CST	58395
UBCJ2	Enzo	ENZ-ABS840
Mouse Anti-rabbit IgG (Conformation Specific)	CST	5127
Universal secondary antibody for avoiding heavy and light chains	Abmart	M21008
HRP-conjugated Goat anti-Rabbit IgG (H+L)	ABclonal	AS014
HRP-conjugated Goat anti-Mouse IgG (H+L)	ABclonal	AS003

Table S3 Primer list.

Primers for RT-PCR		
Gene	Forward primer (5′-3′)	Reverse primer (5′-3′)
FBXO22	CGGAGCACCTTCGTGTTGA	CACACACTCCCTCCATAAGCG
CGB	GAGCTCACCCCAGCATCCTATCACC	TTGATGGGGCGGCACCGTGG
CGA	TGCCCAGAATGCACGCTAC	TTGGACCTTAGTGGAGTGGGA
GCM1	TTCTCCAAGAGTTATGGTCTGGG	CCACGCTTGTAGATCGCCA
ERVW-1	CTGTTGGACTTACTTCACCCAAA	GGTACGGAGGGTTTCATGTAGT
ERVFRD-1	ACCGCCATCCTGATTTCCC	GAGGCTGGATAAGCTGTCCC
CTSD	TGCTCAAGAACTACATGGACGC	CGAAGACGACTGTGAAGCACT
GCM1	TTCCCGGTCACCAACTTCTG	GTAAACTCCCCTGACTTTGTGTT
SDC1	CCACCATGAGACCTCAACCC	GCCACTACAGCCGTATTCTCC
HDAC1	CGCCCTCACAAAGCCAATG	CTGCTTGCTGTACTCCGACA
RCOR1	AACTGGCAAGACGCAGTCAA	GTTGGGCAAATCAGCCAATGA
LSD1	GTGGACGAGTTGCCACATTTC	TGACCACAGCCATAGGATTCC
GAPDH	GGAGCGAGATCCCTCCAAAAT	GGCTGTTGTCATACTTCTCATGG
H2A	CAACGACGAGGAACTGAACAA	ATTTGCTTTTGGCTTTGTGGCTTT
Primers for ChIP-qPCR		
Gene	Forward primer (5′-3′)	Reverse primer (5′-3′)
TEAD4	GCAGTATACACCGAGGCCTC	TCCGTTTTTCTCTCCTGCCC
GATA3	GGTAGAGGAGCGAAGAGGGA	GGCGGGAAAAGAAGACTCCA
SDC1	GGGATTCACCGTGTTAGCCA	GTGGCTCCACAGATCCCTTC
GCM1	ATCAAGCAGGGTTGGTCAGG	GCCCAGGCTGGAGTGTAATT
ERVFRD-1	CCCAGCTCAGGAATGTCAGG	CTGAAGTTGTGCGCATGCTT
CTSD	TGAGGCAGGAGAATCGCTTG	TGCACAGCTGTCTCTTGAGG
CGB7	TGCAACCGAGATGCCTGAAT	CCCTGGCATCCAAATTCCCT
