# Supplementary material for: Reduced FBXO22 skews human trophoblast fate equilibrium toward syncytialization via polyubiquitinating the CoREST complex
Source: Nucleic Acids Res. 2026 Jun 4;54(10):gkag557. doi: 10.1093/nar/gkag557 (PMC13234505; doi:10.1093/nar/gkag557)
Supplement: gkag557_Supplemental_Files [file gkag557_supplemental_files.zip › Supplementary Dataset Legends.docx]

**Supplementary Dataset Legends**

**Supplementary Dataset S1.** Upregulated genes in *FBXO22*-KD hTSCs compared with control hTSCs.

**Supplementary Dataset S2.** RNA-seq analysis of villous tissues from normal pregnancies and RPL patients.

**Supplementary Dataset S3.** FBXO22-interacting proteins identified by IP-MS.

**Supplementary Dataset S4.** List of genes associated with increased H3K27ac enrichment and decreased H3K9me2 enrichment in *FBXO22*-KD hTSCs.

**Supplementary Dataset S5.** Genomic positions of STB-related gene promoter regions tested in luciferase reporter assays.
